# YAP/TEAD4/SP1-induced VISTA expression as a tumor cell-intrinsic mechanism of immunosuppression in colorectal cancer

**DOI:** 10.1038/s41418-025-01446-2

**Published:** 2025-01-28

**Authors:** Zhehui Zhu, Rui Ding, Wei Yu, Yun Liu, Zhaocai Zhou, Chen-Ying Liu

**Affiliations:** 1 Department of Colorectal and Anal Surgery, Xinhua Hospital, Shanghai Jiao Tong University School of Medicine, Shanghai, 200092 China; 2 State Key Laboratory of Genetic Engineering, School of Life Sciences, Zhongshan Hospital, Fudan University, Shanghai, 200438 China; 3 Shanghai Colorectal Cancer Research Center, Shanghai, 200092 China

**Keywords:** Oncogenes, Immunochemistry, Gene regulation

## Abstract

Hyperactivation of the YAP/TEAD transcriptional complex in cancers facilitates the development of an immunosuppressive tumor microenvironment. Herein, we observed that the transcription factor SP1 physically interacts with and stabilizes the YAP/TEAD complex at regulatory genomic loci in colorectal cancer (CRC). In response to serum stimulation, PKCζ (protein kinase C ζ) was found to phosphorylate SP1 and enhance its interaction with TEAD4. As a result, SP1 enhanced the transcriptional activity of YAP/TEAD and coregulated the expression of a group of YAP/TEAD target genes. The immune checkpoint V-domain Ig suppressor of T-cell activation (VISTA) was identified as a direct target of the SP1-YAP/TEAD4 complex and found to be widely expressed in CRC cells. Importantly, YAP-induced VISTA upregulation in human CRC cells was found to strongly suppress the antitumor function of CD8^+^ T cells. Consistently, elevated VISTA expression was found to be correlated with hyperactivation of the SP1-YAP/TEAD axis and associated with poor prognosis of CRC patients. In addition, we found by serendipity that enzymatic deglycosylation significantly improved the anti-VISTA antibody signal intensity, resulting in more accurate detection of VISTA in clinical tumor samples. Overall, our study identified SP1 as a positive modulator of YAP/TEAD for the transcriptional regulation of VISTA and developed a protein deglycosylation strategy to better detect VISTA expression in clinical samples. These findings revealed a new tumor cell-intrinsic mechanism of YAP/TAZ-mediated cancer immune evasion.

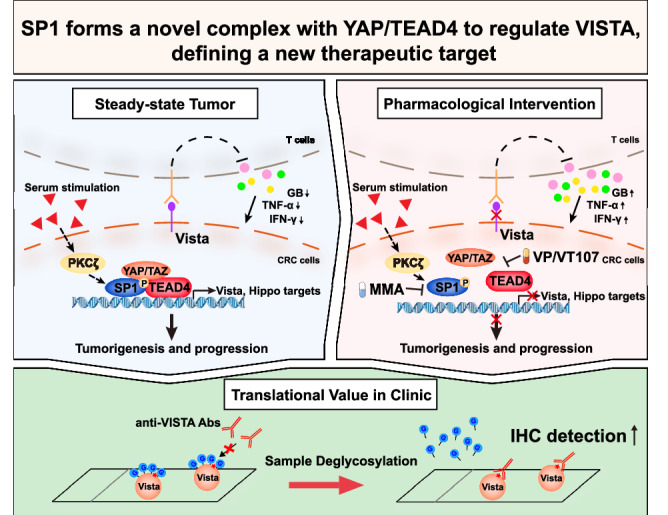

## Introduction

The Hippo pathway has been identified as a key signaling pathway that plays a vital role in controlling organ size and tissue homeostasis [1, 2]. Dysregulation of the Hippo pathway has been widely observed in various cancers, including colorectal cancer (CRC) [3–5]. Activation of YAP/TAZ, the main downstream effectors of the Hippo pathway, modulates the transcription of a series of downstream target genes, mainly through cooperation with TEAD transcription factor members (TEAD1-4), and has been linked to tumor growth, survival, metastasis, and drug resistance [3–6]. Recent studies have also revealed the crucial role of YAP/TAZ in facilitating the development of an immunosuppressive tumor microenvironment (TME) [7–9]. YAP-induced expression of tumor-derived cytokines enhances the recruitment of tumor-infiltrating macrophages, MDSCs and Tregs [10–12]. In addition, YAP/TAZ directly promote the transcription of the *PDL1* gene, which encodes programmed cell death 1 ligand 1 expressed on the tumor cell surface, thus inducing activated T-cell apoptosis and driving immune evasion [13–15]. Although the regulatory axis of YAP/TAZ-PDL1 has been shown in breast, lung and melanoma cancer cell lines, the biological relevance of the YAP/TAZ-PDL1 axis may be dependent on the cell context and has not been studied in traditional syngeneic or genetically engineered mouse models [7–9].

V-domain Ig suppressor of T-cell activation (VISTA), which is encoded by the *VSIR* or *C10ORF54* gene, is a B7 family member that maintains T-cell quiescence and mediates the immunoinhibitory function of myeloid-derived suppressor cells (MDSCs); therefore, VISTA is a promising immune checkpoint target for cancer immunotherapy [16–18]. VISTA has been shown to bind with P-selectin glycoprotein ligand 1 (PSGL-1) and V-set and Ig domain-containing 3 (VSIG3); furthermore, VISTA can function as both a ligand and a receptor to contribute to the immunosuppressive TME [19, 20]. VISTA is highly expressed in lymphoid and myeloid cells. In most cancers, VISTA is predominantly expressed on immune cells in the TME, such as MDSCs, tumor-associated macrophages (TAMs) and tumor-infiltrating lymphocytes (TILs) [16–18]. The expression of VISTA is increased after anti-CTLA4 therapy in patients with prostate cancer and after anti-PD1 therapy in metastatic melanoma patients, which could account for the acquired resistance to immune checkpoint therapy for tumors [21, 22]. Consistently, combination treatment with anti-VISTA and an anti-PD-L1/PD1 mAb has shown enhanced efficacy in a series of in vivo mouse tumor models, including MC38 and CT26 CRC mouse models [20, 23]. A high mRNA level of the VISTA-encoding gene *VSIR* is associated with poor overall survival in CRC patients; however, another study reported that a high protein level of VISTA is an independent favorable prognostic marker for CRC patients [24, 25]. In addition, recent studies have revealed expression of VISTA in tumor cells of ovarian cancer, melanoma and CRC [25–27]. However, it appears that the expression rate of VISTA is much lower in tumors than in immune cells; only 1.8–2.6% of CRC samples have positive staining for tumor cells [25]. More importantly, the biological function of VISTA expression in colorectal cancer cells and the underlying transcriptional mechanism of VISTA have not been determined.

The transcription factor specificity protein 1 (SP1) belongs to the Sp/Kruppel-like factor (KLF) family and recognizes the GC-rich DNA binding motif [28]. Over the past forty years, the role of SP1 in cancer has been well documented in almost every aspect of the “hallmarks of cancer” [28]. Overexpression of SP1 frequently occurs in multiple cancers, including CRC, and is associated with poor prognosis [28]. TFs usually cooperate with each other to synergistically modulate gene transcription [29]. For example, SP1 normally interacts with NF-Y transcription factors, such as TERT, to cooperatively regulate the expression of target genes [28, 30]. Similarly, the YAP/TEAD complex cooperates with other transcription factors, such as AP1, ZEB1, IRF3 and HHEX, to sustain the transcriptional outputs of YAP/TAZ to promote tumorigenesis and tumor progression [31–34].

Recently, we identified that SP1 cooperates with transcription factor ELK4 to modulate a subset of pro-angiogenic genes and promote CRC progression [35]. We noticed that knockdown of SP1 in CRC cells resulted in decreased expression of multiple YAP direct target genes, which prompted us to explore whether SP1 cooperates with YAP/TEAD complex to promote tumorigenesis and tumor progression in CRC. In this study, we report that SP1 interacts with YAP/TEAD, which is enhanced by PKCζ-dependent phosphorylation of SP1. SP1 acts as a positive regulator of the YAP/TEAD complex and to promote its transcriptional activity and protumorigenic function in CRC. Importantly, VISTA was identified as a direct co-target gene of YAP/TEAD and SP1 in CRC, mediating the immunoinhibitory function of YAP-active CRC cells. We showed that VISTA is expressed and glycosylated in CRC cells and that deglycosylation of VISTA greatly improved the sensitivity of detecting the protein level of VISTA by IHC. By using this approach, we found VISTA expression in CRC cells is positively correlated with poor clinical outcomes of CRC patients. Collectively, our study revealed the transcriptional regulatory mechanism of VISTA by the YAP/TEAD-SP1 transcriptional complex in CRC cells, and further highlights YAP/TEAD-SP1-mediated elevation of VISTA as a tumor cell intrinsic mechanism for immune evasion.

## Results

### SP1 enhances the transcriptional activity of the YAP/TEAD complex to promote CRC

**Fig. 1:**
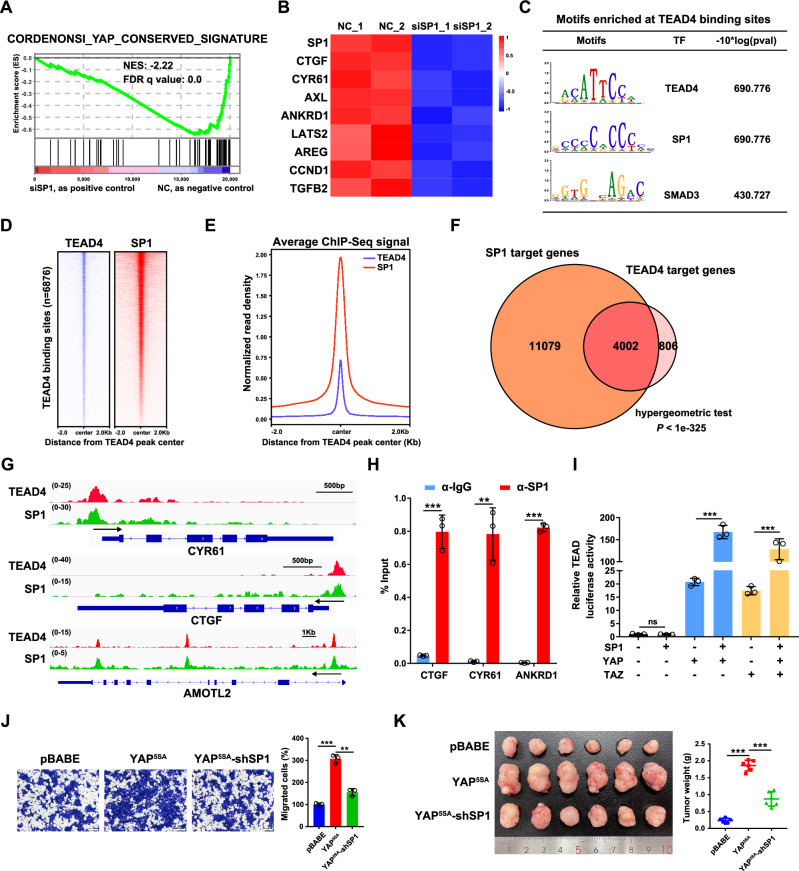
SP1 is a coregulator of TEAD4 in CRC. **A** GSEA revealed significant enrichment of conserved YAP target genes in SP1-knockdown HCT116 cells. **B** Heatmap showing the mRNA levels of SP1 and Hippo target genes in HCT116 cells with SP1 knockdown, as detected by qPCR. The Z score of each sample was calculated and is shown as a heatmap. **C** Motif enrichment analysis of the TEAD4 binding sites in HCT116 cells. **D** Heatmaps of ChIP-seq data for SP1 and TEAD4 in HCT116 cells. **E** Normalized read density for SP1 and TEAD4 is plotted in the region ±2.0 kb from the TEAD4 peak center. **F** Venn diagram displaying the overlap between the SP1 and TEAD4 target genes in HCT116 cells. A hypergeometric test was performed to calculate the statistical significance. **G** Genome browser view of TEAD4 (red) and SP1 (green) ChIP-seq tracks at the CTGF/CYR61/AMOTL2 loci in HCT116 cells. **H** ChIP‒qPCR analysis of SP1 binding at the CTGF/CYR61/ANKRD1 locus in HCT116 cells. **I** A luciferase reporter assay revealed that SP1 was a positive regulator of YAP/TAZ activity. HEK293T cells were transiently transfected with the indicated plasmids and a TEAD-luciferase reporter. **J** Transwell assays of YAP^5SA^-overexpressing HCT116 cells with SP1 knockdown. **K** Representative images of xenograft tumors derived from YAP^5SA^-overexpressing HCT116 cells with SP1 knockdown are shown.

In our previous study, to elucidate the cooperative mechanism of transcriptional regulation by the ELK4-SP1/3 complex, we performed an RNA-seq analysis in SP1/3-knockdown HCT116 cells [35]. Intriguingly, the mRNA levels of multiple classic YAP/TEAD target genes were downregulated in SP1- but not SP3- knockdown HCT116 cells, which was further supported by the gene set enrichment analysis (GSEA) (Fig. 1A). The results of qPCR analysis confirmed that the mRNA levels of classic YAP/TAZ target genes, including CTGF, CYR61, AXL, ANKRD1, LATS2, AREG, CCND1 and TGFB2, were decreased upon the knockdown of SP1 in HCT116 cells (Fig. 1B). Similar results were observed in two other CRC cell lines (Supplementary Fig. S1A).

Next, we explored whether genomic occupancy profiles of TEAD4 and SP1 overlap in CRC cells by analyzing publicly available ChIP-seq datasets for TEAD4 and SP1 in HCT116 cells from the ENCODE database [36]. The results of motif enrichment analysis revealed that, in addition to the well-known AP1 binding motif, the SP1 binding motif was also enriched in TEAD4 binding sites and vice versa (Fig. 1C and Supplementary Fig. S1B). Significantly, genomic landscape analysis revealed that SP1 was enriched in regions surrounding TEAD4-binding sites (Fig. 1D, E). This analysis indicated that a total of 4002 genes were coregulated by TEAD4 and SP1 in HCT116 cells; these genes account for 83.2% of the target genes of TEAD4 and include the classic target genes of YAP/TEAD (CTGF, CYR61, and AMOTL2) (Fig. 1F, G). Further ChIP-qPCR assay confirmed that SP1 bound to the promoter regions of CTGF, CYR61 and ANKRD1 (Fig. 1H). In addition, a luciferase reporter assay showed that overexpression of SP1 augmented the YAP/TAZ-induced activation of TEAD (Fig. 1I).

Both YAP and SP1 play vital roles in tumorigenesis and tumor progression. To determine the functional interplay between YAP and SP1, we established HCT116 cell lines stably expressing a constitutively active YAP mutant, namely YAP^5SA^, with or without SP1 shRNA (Supplementary Fig. S1C). Knockdown of SP1 attenuated the increase in the mRNA expression of CTGF, CYR61 and ANKRD1 (Supplementary Fig. S1D), suggesting that SP1 is required for YAP-mediated gene transcription and that SP1 regulation of YAP is independent of upstream Hippo signaling. Functionally, the YAP^5SA^ overexpression increased cell proliferation and migration in vitro and promoted tumor growth in vivo; while such effects were significantly abrogated by SP1 knockdown (Fig. 1J, K, Supplementary Fig. S1E, F). Together, these data suggest that the transcription factor SP1 could be a transcriptional coregulator of YAP/TEAD4, which cooperatively promote tumorigenesis and tumor progression in CRC.

### SP1 interacts with the YAP/TEAD complex

**Fig. 2:**
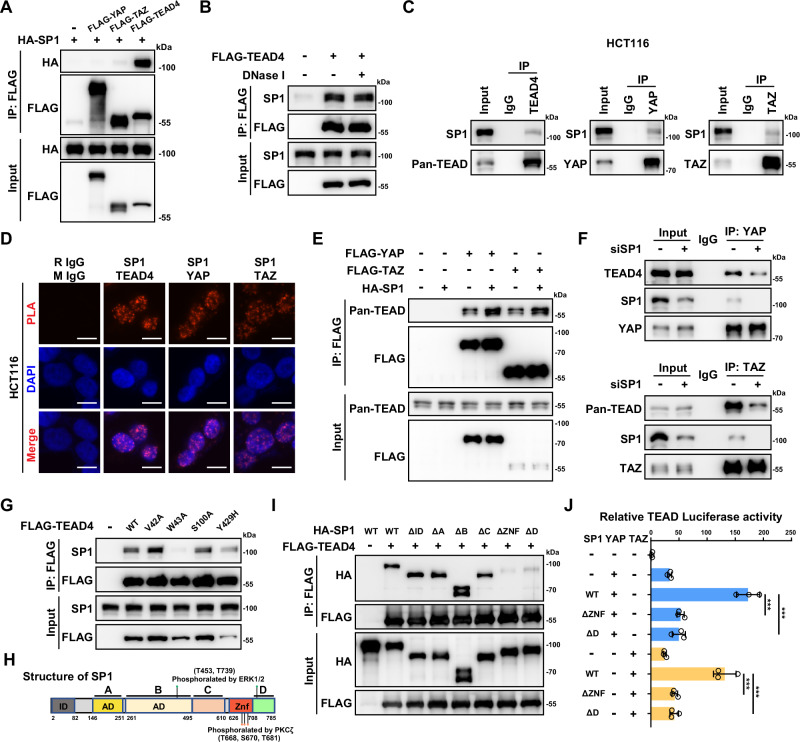
SP1 interacts with the YAP/TEAD4 complex. **A** Coimmunoprecipitation (co-IP) was used to evaluate the interactions between exogenous FLAG-YAP/TAZ/TEAD4 and HA-SP1 in HEK293T cells. **B** Co-IP of exogenous FLAG-TEAD4 and endogenous SP1 in HCT116 cells in the presence or absence of DNase I. **C** Co-IP of endogenous SP1 with YAP, TAZ and TEAD4 in HCT116 cells. **D** Proximity ligation assay (PLA) showing the interactions between endogenous SP1 and YAP, TAZ and TEAD4 in HCT116 cells. **E** SP1 enhances the interactions between YAP/TAZ and TEADs. Semiendogenous co-IP of exogenous FLAG-YAP, FLAG-TAZ and endogenous TEADs in HCT116 cells with or without HA-SP1 overexpression. **F** Co-IP of endogenous YAP, TAZ and TEADs in HCT116 cells with or without SP1 knockdown. **G** The TEAD4-W43A mutant abolished the interaction between exogeneous TEAD4 and endogenous SP1. HCT116 cells were transfected with wild-type TEAD4 and various TEAD4 mutant plasmids as indicated. **H** Domain organization maps of the full-length SP1 protein and its phosphorylation sites by ERK1/2 and PKCζ. **I** Co-IP was performed to map the domains mediating the interactions between SP1 and TEAD4 in HCT116 cells. **J** Luciferase reporter assays showing that the ZNF and D domains of SP1 are required for YAP/TAZ activation by SP1.

The transactivation of YAP/TEAD by SP1 in a manner independent of the upstream Hippo signaling prompted us to explore the potential interaction between SP1 and the YAP/TEAD complex. Upon exogenous expression of SP1/YAP/TAZ/TEAD4 in 293 T cells, we observed strong interactions of SP1 with TEAD4 but not YAP/TAZ (Fig. 2A). Furthermore, we confirmed that all four members of the TEAD transcription factor family could interact with SP1 (Supplementary Fig. S2A). Since the four members of the TEAD transcription factor are functionally redundant and TEAD4 is the only TEAD family member overexpressed in CRC [37], we focused on TEAD4 as the representative of TEAD family members in this study. DNase I treatment did not affect the interaction between TEAD4 and SP1, further indicating DNA-independent nature of the SP1/TEAD interaction (Fig. 2B). Next, we tested the interaction between SP1 and YAP/TAZ/TEAD in HCT116 CRC cells. In contrast to the co-IP result of the exogenous proteins in 293 cells, our co-IP in HCT116 cells indicated that endogenous proteins of YAP, TAZ and TEAD can all mildly interact with SP1 (Fig. 2C). A subsequent proximity ligation assay (PLA) in HCT116 cells confirmed the interaction of endogenous SP1 with endogenous YAP, TAZ and TEAD4 (Fig. 2D). Interestingly, although the interaction between exogenous YAP/TAZ and SP1 was quite weak, we observed that overexpression of SP1 promoted the formation of the YAP/TAZ-TEAD complex in 293 T cells (Fig. 2E). Consistently, knockdown of SP1 moderately attenuated the interaction of endogenous TEAD protein with YAP/TAZ in HCT116 cells, indicating that SP1 could stabilize the YAP/TEAD complex (Fig. 2F).

Recently, we identified HHEX as a transcriptional coregulator of YAP/TEAD4 [34]. We utilized previously constructed TEAD4 DNA binding domain mutants and tested the potential effect of these mutations on the assembly of SP1-TEAD4 complex. Intriguingly, only the W43A mutation almost completely disrupted the interaction between SP1 and TEAD4 (Fig. 2G). However, all three mutations in the TEAD4 DNA binding domain diminished the HHEX-TEAD4 interaction, suggesting that TEAD4 might interact with different transcription factors through different interfaces [34]. We also mapped the specific domain of SP1 that mediate its interaction with TEAD4. As shown in Fig. 2I, deletion of either the ZNF domain or the C-terminal D domain of SP1 dramatically attenuated the interaction of SP1 with TEAD4, suggesting that the C-terminal domain of SP1 is vital for the interaction between SP1 and TEAD4 (Fig. 2H). In line with the above observation, both the loss of the ZNF domain and the loss of the C-terminal D domain led to largely abolished SP1-induced transactivation of TEAD (Fig. 2J). Collectively, these data indicate that SP1 could interact with and enhance the transcriptional activity of the YAP/TEAD complex.

### Serum stimulates SP1-TEAD4 interaction dependent on PKCζ kinase

**Fig. 3:**
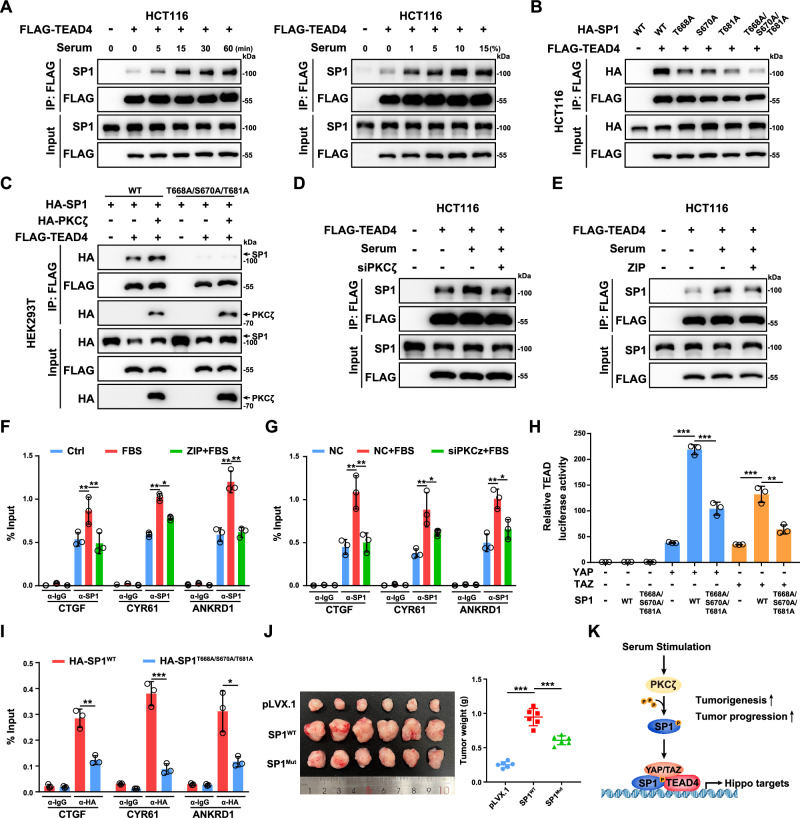
PKCζ facilitates the interaction between SP1 and TEAD4. **A** Serum stimulation enhanced the interaction between SP1 and TEAD4 in HCT116 cells. HCT116 cells were transfected with the indicated plasmids and serum-starved overnight. Then, the cells were treated with 10% serum for the indicated times or with the indicated serum concentration for 15 min. **B** Co-IP analysis of the interactions between TEAD4 and WT or mutant SP1 in HCT116 cells. **C** PKCζ enhanced the interaction between TEAD4 and WT SP1 but not between TEAD4 and the phosphodeficient mutant SP1. **D** Knockdown of PKCζ by siRNA abrogated the serum-induced interaction between SP1 and TEAD4 in HCT116 cells. **E** The PKCζ inhibitor ZIP abrogated the serum-induced interaction between SP1 and TEAD4 in HCT116 cells. The cells were pretreated with 1 μM ZIP for 12 h and then stimulated with 10% serum for 15 min. **F** ChIP‒qPCR analysis of SP1 binding to TEAD binding sites at the CTGF, CYR61 and ANKRD1 genomic loci in HCT116 cells. The cells were pretreated with or without 1 μM ZIP for 12 h and then stimulated with 10% serum for 15 min. **G** ChIP‒qPCR analysis of SP1 binding to TEAD binding sites at the CTGF, CYR61 and ANKRD1 genomic loci in control and PKCζ knockdown HCT116 cells. Cells were transfected with siRNA to knock down PKCζ for 48 h and then stimulated with 10% serum for 15 min. **H** Luciferase reporter assay showing the decrease in YAP/TAZ activation by the T668A/S670A/T681A mutant SP1. HEK293T cells were transiently transfected with the indicated plasmids and a TEAD-luciferase reporter. **I** ChIP‒qPCR analysis of SP1 binding to TEAD binding sites at the CTGF, CYR61 and ANKRD1 genomic loci in HCT116 cells stably expressing WT or the T668A/S670A/T681A mutant of SP1. **J** Representative images of xenograft tumors derived from HCT116 cells overexpressing WT or the T668A/S670A/T681A mutant SP1 are shown. **K** Schematic illustration showing that PKCζ phosphorylates SP1 and subsequently promotes the activation of the SP1-TEAD4-YAP/TAZ transcriptional complex in CRC cells under serum stimulation.

Serum is a well-known stimulator of YAP/TEAD transcriptional activity through inhibiting the upstream kinase LATS1/2 [38]. However, the knockdown of LATS1/2 showed minimal effect on the formation of SP1-TEAD4 complex in HCT116 cells (Supplementary Fig. S3A). Meanwhile, serum can induce SP1 phosphorylation and lead to enhanced transcription of direct target genes of SP1 [39, 40]. Thus, we explored whether serum could modulate the interaction between SP1 and TEAD4 in CRC cells. Co-IP assays showed that serum treatment enhanced the interaction between SP1 and TEAD4 in HCT116 cells (Fig. 3A). Since either the ZNF domain or the C-terminal D domain of SP1 mediates the SP1-TEAD4 interaction and these two domains are phosphorylated by PKCζ and ERK1 upon serum treatment, we generated a series of SP1 constructs with its phosphorylation site mutated [40] (Fig. 2H). Mutation of the ERK1 phosphorylation sites (T453 and T739) slightly decreased the SP1-TEAD4 interaction (Supplementary Fig. S3B). Intriguingly, individual mutations of PKCζ phosphorylation sites (T668, S670 and T681) moderately decreased the SP1-TEAD4 interaction, and simultaneous mutation of three PKCζ phosphorylation sites dramatically abrogated SP1-TEAD4 complex formation (Fig. 3B). Consistently, overexpression of PKCζ moderately increased the interaction between SP1 and TEAD4 (Fig. 3C). Notably, PKCζ also interacted with TEAD4 in CRC cells (Fig. 3C and Supplementary Fig. S3C). In addition, knockdown of PKCζ or treatment with the PKCζ inhibitor ZIP attenuated the serum-induced interaction between SP1 and TEAD4, recruitment of SP1 to the genomic locus and upregulation of the mRNA levels of the YAP/TEAD target genes (Fig. 3D-G, Supplementary Fig. S3D and S3E). Similarly, a TEAD reporter assay showed that the T668A/S670A/T681A triple mutation significantly attenuated SP1-induced TEAD activation (Fig. 3H). Importantly, ChIP–qPCR results further showed decreased occupancy of SP1^T668A/S670A/T681A^ on the TEAD4-bound genomic regions of CTGF, CYR61 and ANKRD1 (Fig. 3I).

Next, we generated HCT116 cell lines stably expressing WT or T668A/S670A/T681A mutant SP1 to explore the effect of SP1 phosphorylation by PKCζ on the protumorigenic function of SP1. Compared to WT SP1, overexpression of T668A/S670A/T681A mutant attenuated the promoting effect of SP1 on the transcription of YAP/TEAD target genes (Supplementary Fig. S3F). By using CCK8 and Transwell assays, we observed that HCT116 cells expressing the T668A/S670A/T681A mutant of SP1 had decreased proliferation and cell migration ability (Supplementary Fig. S3G and S3H). A xenograft assay further confirmed the effect of the T668A/S670A/T681A mutation diminishing the protumorigenic function of SP1 (Fig. 3J and Supplementary Fig. S3I). In addition, the PKCζ inhibitor ZIP largely attenuated the enhanced cell proliferation and cell migration induced by overexpression of YAP-5SA in HCT116 cells (Supplementary Fig. S3J and S3K). Collectively, our data indicate that PKCζ positively regulates the interaction between SP1 and TEAD4 and that SP1 cooperates with TEAD4 to promote tumorigenesis in CRC (Fig. 3K).

### Mithramycin A and verteporfin synergistically suppress CRC growth

**Fig. 4:**
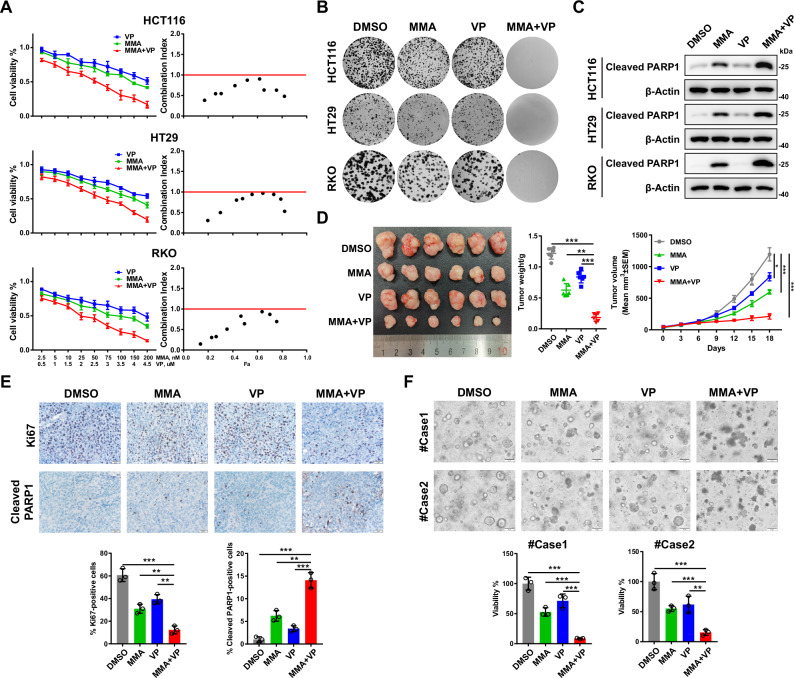
Mithramycin A in combination with verteporfin has synergistic antitumor effects on CRC. **A** Cell viability was assessed following 48 h of exposure to the indicated concentrations of MMA or VP alone or in combination in HCT116, HT29 and RKO cells (*n* = 4 biologically independent samples per group). CI (combination index) values for the various combinations were calculated using CompuSyn. A CI < 1.0 indicates a synergistic effect. **B** Colony formation assays showing the synergistic antitumor effects of the combination of MMA (100 nM) and VP (2 μM) on HCT116, HT29 and RKO cells. **C** Representative western blots of cleaved PARP1 in HCT116, HT29 and RKO cells treated with MMA (100 nM) or VP (2 μM) alone or in combination for 24 h. **D** Representative images of HCT116 cell-derived xenografts harvested from nude mice treated with MMA and VP alone or in combination (*n* = 6 mice per group) are shown (left). The tumors were weighed (middle), and growth curves were plotted (right). **E** Representative images of IHC staining for Ki67 and cleaved PARP1 in HCT116 cell-derived xenografts harvested from nude mice treated with MMA or VP alone or in combination (scale bars = 20 μm). **F** Representative images of two CRC PDOs treated with MMA (100 nM) or VP (2 μM) alone or in combination for 48 h, and cell viability was evaluated with a CellTiter-Glo assay.

Our finding that SP1 cooperates with the YAP/TEAD complex to regulate gene transcription in CRC raises the possibility that combination of an SP1 inhibitor with a YAP/TEAD inhibitor might synergistically suppress CRC growth. To test this hypothesis, we treated three CRC cell lines (HCT116, HT29, and RKO) with the SP1 inhibitor mithramycin A (MMA) and the YAP/TEAD inhibitor verteporfin (VP). Both CCK-8 and colony formation assays showed that cotreatment with MMA and VP inhibited cell proliferation in the three CRC cell lines in a stronger extent than treatment with a single agent (Fig. 4A, B). The combination index (CI) was less than 1, indicating a synergistic inhibitory effect of MMA in combination with VP on CRC growth (Fig. 4A). Furthermore, western blot analysis of the apoptotic marker cleaved PARP1 showed that VP had a minor effect on apoptosis in HCT116, HT29 and RKO cells, while cotreatment with MMA and VP induced more cell apoptosis than treatment with MMA alone (Fig. 4C).

Next, we explored the synergistic effect of cotreatment with MMA and VP in an HCT116 cell line-derived xenograft tumor model. Treatment with MMA or VP alone or cotreatment showed minimal effect on the mice body weight (Supplementary Fig. S4A). Consistent with the in vitro observations, cotreatment with MMA and VP more dramatically suppressed tumor growth than treatment with a single agent, which was in agreement with the greater decrease in the number of Ki67-positive cells and increase in cell apoptosis (Fig. 4D, E). We also assessed the synergistic suppressive effect of MMA and VP in two patient-derived CRC organoids. Similarly, single-agent treatment with MMA or VP led to mild inhibition of organoid growth, while cotreatment with MMA and VP significantly decreased the viability of the CRC organoids (Fig. 4F). The synergistic anti-tumor effect of SP1 inhibitor and TEAD inhibitor was also observed by using a newly developed pan-TEAD inhibitor of TEAD auto-palmitoylation, VT107 [41] (Supplementary S4B–F). Taken together, these results indicate a synergistic antitumor effect for targeting SP1 and YAP/TEAD in CRC.

### YAP/TEAD/SP1 cooperatively activates the transcription of VISTA in CRC cells

**Fig. 5:**
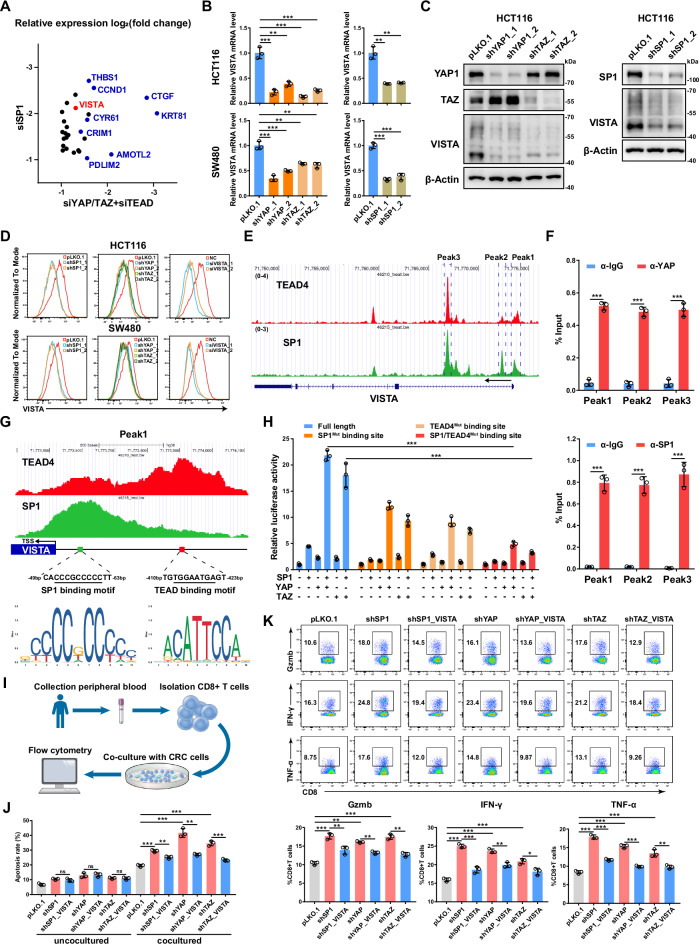
VISTA is transcriptionally activated by the SP1-YAP/TAZ/TEAD4 complex in CRC. **A** Scatter plot showing a set of genes differentially expressed by both SP1 knockdown and YAP/TAZ/TEAD knockdown in HCT116 cells (log_2_FC < -1, *P* < 0.05). **B** qPCR analysis of the VISTA mRNA level in HCT116 and SW480 cells with YAP, TAZ or SP1 knockdown. **C** Western blot analysis of the protein level of VISTA in HCT116 cells with YAP, TAZ or SP1 knockdown. **D** VISTA expression was detected by flow cytometry in HCT116 and SW480 cells with YAP, TAZ or SP1 knockdown. Knockdown of VISTA in HCT116 and SW480 cells by siRNAs was used as a positive control. **E** Genome browser view of TEAD4 (red) and SP1 (green) ChIP-seq tracks at the VISTA gene locus. **F** ChIP‒qPCR analysis of YAP (top) and SP1 (bottom) binding to the VISTA genomic locus in HCT116 cells. **G** Genome browser view of TEAD4 (red) and SP1 (green) ChIP-seq tracks of the promoter of VISTA. **H** The relative luciferase activities of the full-length, SP1-mutant and TEAD4-binding site-mutant VISTA promoters were detected in HEK293T cells transfected with the indicated plasmids. **I** Schematic representation of the coculture system of CRC cells and CD8^+^ T cells. **J** Cell apoptosis was analyzed by flow cytometry in SP1-, YAP- or TAZ-knockdown HCT116 cells cultured alone or cocultured with CD8^+^ T cells. **K** CD8^+^ T cells were cocultured with SP1-, YAP- or TAZ-knockdown HCT116 cells with or without VISTA overexpression for 48 h. Gzmb, IFN-γ and TNF-α expression was assessed via flow cytometry.

By integrating the RNA-seq data of YAP/TAZ, TEAD1/2/3/4 and SP1 knockdown HCT116 cells, we further analyzed the downstream target genes coregulated by YAP/TAZ/TEAD and SP1 in CRC cells (Fig. 5A). In addition to the well-known target genes of YAP/TAZ, such as CTGF, CYR61, AMOTL2, CRIM1, CCND1 and THBS1, we noted VISTA but not PDL1. We then confirmed that knockdown of YAP, TAZ and SP1 decreased the mRNA and protein expression of VISTA in HCT116 and SW480 cells (Fig. 5B, C and Supplementary Fig. S5A). To further verify the expression of VISTA in CRC cells, we performed flow cytometry analysis and found that knockdown of VISTA significantly decreased its protein level on the cell membrane of CRC cells (Fig. 5D and Supplementary Fig. S5B). Moreover, the protein level of VISTA on the cell membrane decreased in HCT116 and SW480 cells following individual knockdown of YAP, TAZ or SP1 (Fig. 5D). The mRNA level of VISTA was also downregulated in HCT116 and RKO cells treated with SP1 inhibitor MMA and TEAD inhibitor VT107 (Supplementary Fig. S5C). In contrast, overexpression of a constitutively active mutant YAP, i.e., YAP^5SA^ but not a version defective in binding TEAD, i.e., YAP^5SA-^S94A enhanced the expression of VISTA in HCT116 and SW480 cells (Supplementary Fig. S5D, E). Furthermore, overexpression of WT SP1, but not its T668A/S670A/T681A mutant increased VISTA mRNA expression in HCT116 cells (Supplementary Fig. S5F). Meanwhile, knockdown of the SP1 upstream kinase PKCζ significantly decreased the mRNA level of VISTA in HCT116 and SW480 cells (Supplementary Fig. S5G).

Next, we explored the molecular mechanism through which VISTA gene transcription is coregulated by YAP/TEAD and SP1. Public ChIP-seq data for TEAD4 and SP1 in HCT116 cells revealed multiple TEAD4 and SP1 cobound peaks across the VISTA genomic locus from the promoter to the second intron, which was further confirmed by ChIP‒qPCR (Fig. 5E, F). Notably, the TEAD4 peak with lower intensity in the VISTA promoter was found to be adjacent to the TSS and overlapped with the SP1 peak with high intensity, while the second TEAD4 peak with higher intensity was found to be located within 350 bp of the overlapping peak of TEAD4/SP1 (Fig. 5G). The SP1 binding motif in the TEAD4/SP1 overlapping peak and the TEAD binding motif in the TEAD4 peak were found to have greater intensities (Fig. 5G). These data suggested that TEAD4 and SP1 cooperatively bind to and modulate VISTA gene transcription. A subsequent VISTA promoter luciferase reporter assay revealed mild to moderate activation of the reporter following individual overexpression of YAP, TAZ and SP1, and the coexpression of YAP/TAZ and SP1 dramatically enhanced the luciferase activity of the VISTA promoter; however, this effect was largely abolished by mutation of the SP1 binding motif or TEAD binding motif in the SP1 promoter (Fig. 5H). In addition, the truncated SP1 variant (with deletion of the ZNF domain or the C-terminal D domain) with a TEAD4 binding defect lost the ability to cooperatively activate the VISTA promoter reporter (Supplementary Fig. S5H).

To explore the pathological relevance of the YAP/TEAD/SP1-VISTA axis in CRC, we established a system to co-culture HCT116 cells with activated human CD8^+^ T cells isolated from the peripheral blood of healthy donors (Fig. 5I). Knockdown of SP1 or YAP/TAZ induced mild cell apoptosis in HCT116 cells. Coculture with CD8^+^ T cells significantly increased the apoptosis of HCT116 cells; while knockdown of either SP1 or YAP/TAZ in HCT116 cells further enhanced such effect; moreover, expression of VISTA in HCT116 cells abrogated CD8^+^ T cell-induced apoptosis (Fig. 5J). Consistently, coculture with SP1-knockdown or YAP/TAZ-knockdown HCT116 cells increased the expression of GZMB, IFN-γ and TNF-α in CD8^+^ T cells, and expression of VISTA in SP1- or YAP/TAZ-knockdown HCT116 cells restored the activation of CD8^+^ T cells to basal level (Fig. 5K). In addition, overexpression of YAP^5SA^ in HCT116 and SW480 cells suppressed both intrinsic apoptosis and CD8^+^ T-cell-induced cell apoptosis. However, knockdown of VISTA in HCT116 and SW480 cells expressing YAP^5SA^ abrogated CD8^+^ T-cell-induced cell apoptosis but had no effect on intrinsic apoptosis (Supplementary Fig. S5I). Consistent with these observations, coculture with YAP^5SA^-expressing HCT116 or SW480 cells downregulated the expression of GZMB, IFN-γ and TNF-α in CD8^+^ T cells, and knockdown of VISTA in YAP^5SA^-expressing HCT116 and SW480 cells led to increased activation of CD8^+^ T cells (Supplementary Fig. S5J). Taken together, these data support that YAP/TEAD/SP1 could endow CRC cells with resistance to T-cell-mediated cytotoxicity through upregulation of VISTA expression.

### Deglycosylation of VISTA enhances the sensitivity of IHC detecting VISTA

**Fig. 6:**
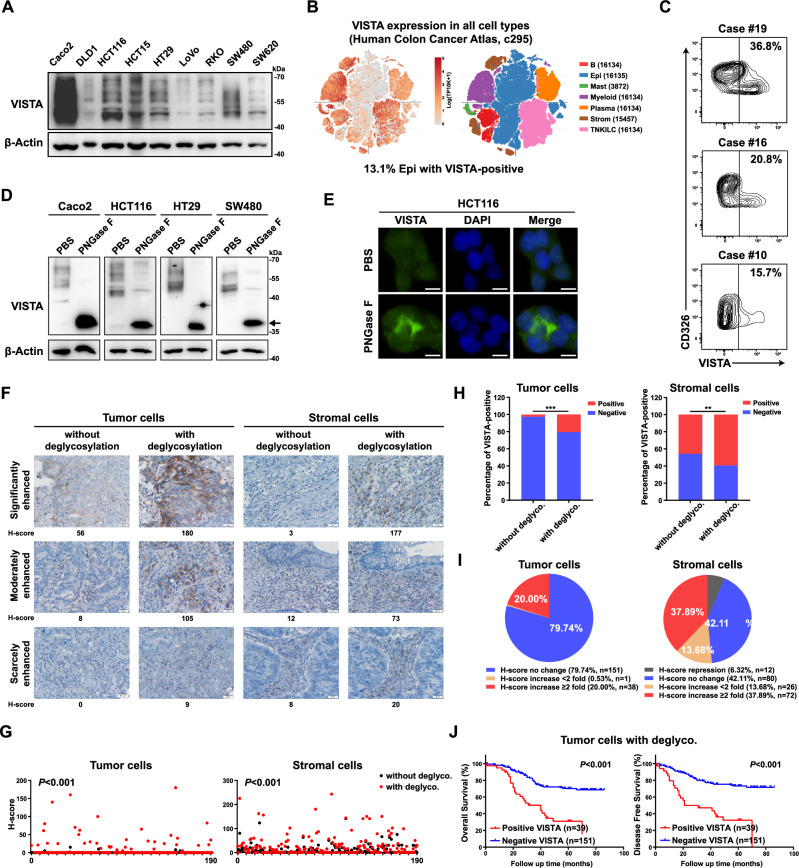
Removal glycosylation enhances VISTA detection. **A** Western blot analysis of the VISTA protein in a panel of CRC cell lines. **B** Single-cell analysis of VISTA mRNA expression in CRC tissues. **C** Flow cytometry analysis of VISTA protein expression in primary CRC tissues. Cells were gated for CD326 positivity to select epithelial cells, and the VISTA expression level was determined and analyzed in this subset compared to that in negative controls. **D** Lysates of CRC cell lines were processed with or without deglycosylation by PNGase F (5%) pretreatment and probed for VISTA expression by western blotting. The arrow indicates nonglycosylated VISTA. **E** VISTA expression was detected by immunofluorescence in HCT116 cells treated with or without deglycosylation by PNGase F (5%) pretreatment (scale bars = 5 μm). Representative images (**F**) and H-scores (**G**) of VISTA IHC staining of tumor cells and stromal cells in CRC tissues processed with or without deglycosylation by PNGase F (5%) pretreatment (scale bars = 20 μm). The percentage of VISTA-positive cells (**H**) and pie charts highlighting the fold change in the H-score (**I**) are shown. **J** K‒M plots of overall survival (left) and disease-free survival (right) of CRC patients stratified by the IHC signal intensity of VISTA in tumor cells. A CRC tissue array (TMA, *n* = 190) was subjected to deglycosylation by PNGase F (5%) pretreatment before regular IHC analysis.

As a novel immune checkpoint molecule, VISTA is reportedly expressed primarily in hematopoietic and myeloid cells [16–18]. Based on our finding of the YAP/TEAD/SP1-VISTA axis, we further examined VISTA expression in a panel of CRC cells. Strikingly, moderate to strong protein levels of VISTA were observed in all CRC cell lines (Fig. 6A). By exploring the DepMap database and combining gene expression data from the Cancer Cell Line Encyclopedia (CCLE) [42], we found that the mRNA level of VISTA in CRC cells was comparable to the mRNA level in ovarian cancer and melanoma cells, which was recently reported to be associated with VISTA expression [26, 27] (Supplementary Fig. S6A). Notably, high VISTA mRNA levels were also detected in other types of cancers, including bile duct cancer, bladder cancer, lung cancer and prostate cancer (Supplementary Fig. S6A). Here, we found that VISTA is expressed in both A549 lung cancer cells and DU145 prostate cancer cells and that its expression was also decreased by YAP/TAZ knockdown (Supplementary Fig. S6B). In addition to the CCLE database, we analyzed a published CRC single-cell sequence dataset comprising 371,223 cells from colorectal tumors and adjacent normal tissues and found that 13.1% of the epithelial cells were VISTA-positive cells [43, 44] (Fig. 6B). To further confirm the presence of VISTA in colorectal epithelial and/or cancer cells, we detected VISTA expression in 20 fresh CRC tissues by FACS and found that 15.7%-36.8% of the CD326^+^ epithelial cells had VISTA expression (Fig. 6C and Supplementary Fig. S6C).

Previously, immunohistochemical (IHC) analysis revealed that VISTA is minimally expressed in CRC tumor cells [25, 45]. Our finding that VISTA was expressed in CRC cells prompted us to speculate that the routine IHC protocol was not suitable for detecting VISTA expression. Inspired by a recent study that removal of N-linked glycosylation enhanced PD-L1 detection in clinical samples [46], we explored whether removing the glycan moieties on VISTA could fully expose its epitope and thus improve the sensitivity of IHC detection. Western blot analysis of VISTA from cell lysates revealed multiple bands ranging from 40 to 70 kDa, indicating glycosylation of VISTA; treatment with peptide-N-glycosidase F (PNGase F), which removes global N-linked glycosylation products, dramatically decreased the molecular weight of the VISTA protein with a homogeneous pattern of ~36 kDa in CRC cell lines (Fig. 6D). Similar results were observed for A549 lung cancer cells and DU145 prostate cancer cells (Supplementary Fig. S6D).

To further validate the glycosylation status of VISTA, HCT116 cells were treated with the *N*-linked glycosylation inhibitor tunicamycin (TM), which dramatically suppressed the glycosylation of VISTA (Supplementary Fig. S6E). Moreover, treatment with TM or the oligosaccharyltransferase inhibitor NGI-1 retained VISTA in the cytoplasm and increased its immunofluorescence signals (Supplementary Fig. S6F). Interestingly, IF analysis of PNGase F before fixation also revealed enhanced immunofluorescence signals for VISTA (Fig. 6E). Next, we assessed the effect of PNGase F treatment on VISTA expression in a CRC tissue array by IHC analysis. Consistent with the findings of previous studies, IHC analysis by the commonly used protocol mainly revealed VISTA expression in stromal cells, with only 5/190 samples showing low levels of VISTA expression in tumor cells (Fig. 6F-H). However, IHC analysis with pre-deglycosylation revealed much greater VISTA expression (Fig. 6F–H). As determined by the histoscore (H-score) [46], the VISTA H-score in tumor cells was increased more than twofold in 20.0% of the samples (Fig. 6I). Similarly, the expression of VISTA in stromal cells was also found to be much higher when the sample was deglycosylated before IHC (Fig. 6F–I).

In addition, we further evaluated the clinical relevance of the VISTA H-score after deglycosylation. Kaplan‒Meier analysis indicated that a positive VISTA signal after deglycosylation in tumor cells was a marker of poor prognosis in CRC patients and was correlated with shorter overall survival (OS) and disease-free survival (DFS) (Fig. 6J). In contrast to the findings of previous reports, we did not observe the prognostic value of VISTA expression in stromal cells detected by routine IHC analysis in our CRC cohort (Supplementary Fig. S6G). However, a positive VISTA signal after deglycosylation in stromal cells was also associated with poor prognosis in CRC patients (Supplementary Fig. S6G). Taken together, these data suggested that N-linked glycosylation of VISTA impedes its detection by IHC and that deglycosylation of VISTA could significantly improve the sensitivity of IHC detection.

### The expression of YAP/SP1 is positively correlated with VISTA expression and indicates poor prognosis in CRC

**Fig. 7:**
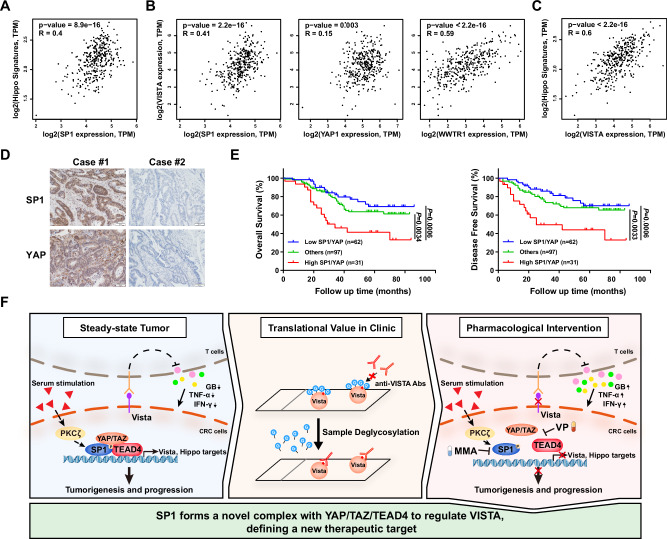
Clinical relevance of the SP1/TEAD4/YAP/TAZ-VISTA axis in CRC. **A** Pearson correlation analysis showing a positive correlation between SP1 expression and the YAP target gene signature in CRC. **B** There was a positive correlation between the expression of SP1/YAP/TAZ and that of VISTA in CRC. **C** Pearson correlation analysis revealed a positive correlation between VISTA expression and the YAP target gene signature in CRC. **D** Representative images of IHC staining for SP1 and YAP in CRC tissues. **E** K‒M Analysis of overall survival (left) and disease-free survival (right) in CRC patients stratified by the protein levels of SP1 and YAP. **F** A schematic illustration of our findings that PKCζ activates the formation of the SP1-YAP/TAZ/TEAD4 complex to transcriptionally regulate VISTA and that protein deglycosylation enhances VISTA detection by IHC in CRC.

Finally, we analyzed data from the publicly available TCGA CRC dataset and found that the mRNA level of SP1 was positively correlated with the mRNA levels of classical YAP/TEAD target genes, such as CTGF, CYR61, and AXL, as well as the YAP signature (7 classical YAP/TEAD target genes) in CRC (Fig. 7A and Supplementary Fig. S7A). Consistent with these findings, the mRNA level of VISTA was found to be positively correlated with the mRNA levels of YAP, TAZ and SP1, as well as with the mRNA levels of classical YAP/TEAD target genes and the YAP signature (Fig. 7B, C and Supplementary Fig. S7B). Similar results were obtained in the 76 CRC cell lines by analyzing the gene expression profiles from the CCLE datasets (Supplementary Fig. S7C). Both YAP and SP1 are overexpressed and have been reported to be associated with poor prognosis in CRC. In addition, 16.3% of the CRC samples highly expressed both YAP and SP1, and the corresponding patients had the worst OS and DFS rates (Fig. 7D, E). Taken together, these results demonstrated that upregulation of SP1 is associated with YAP/TEAD transcriptional activity, and such upregulation of SP1 could dampen the antitumor immunity through the transcriptional activation of VISTA in CRC (Fig. 7F).

## Discussion

SP1 is a ubiquitously expressed, prototypic C2H2-type zinc finger-containing transcription factor that was originally identified as a transcription factor that activates the early and late simian virus 40 (SV40) promoters [47]. Moreover, TEAD1 was originally known as transcriptional enhancer factor 1 (TEF1) and was found to stimulate the transcription of the early and late SV40 promoters through binding to SV40 enhancers, which suggests the potential cooperative role of SP1 and TEAD1 in regulating SV40 promoters [48]. Hyperactivation of both YAP/TEAD and SP1 is commonly observed in human cancers, and multiple transcription factors have been reported to cooperate with YAP/TEAD or SP1 to drive tumorigenesis and tumor progression [1, 3, 5, 28]. In this study, we discovered that SP1 interacts and cooperates with the YAP/TEAD complex to activate gene transcription in CRC. We found that SP1 stabilizes the YAP/TEAD complex in CRC. Interestingly, SP1 primarily binds to TEAD4 but not YAP/TAZ in 293 T cells, indicating context-dependent assembly of the YAP/TEAD/SP1 transcriptional complex and preferential binding of SP1 to TEAD4. Consistently, the YAP-binding-defective TEAD4 mutant was found to still readily bind to SP1, suggesting a direct interaction between TEAD4 and SP1. Notably, our previous study showed that several mutations in the DNA-binding TEA domain of TEAD4 could abolish its interaction with HHEX; here we found only one of those mutations abrogated the TEAD4-SP1 interaction. Thus, TEAD4 can interact with different transcription factors, such as SP1 and HHEX, through different interfaces.

PKCζ can bind and phosphorylate the zinc finger region of SP1 [40]. PKCζ-dependent SP1 phosphorylation is involved in VEGF expression in RCC cancer cells [49, 50]. Although the sites phosphorylated by PKCζ are located in the DNA-binding domain of SP1, mutation of PKCζ-dependent phosphorylation sites does not perturb the DNA-binding ability of SP1 [49, 50]. Our discovery of the PKCζ-SP1-TEAD4 regulatory axis suggested that PKCζ-dependent SP1 phosphorylation could enhance the interaction between SP1 and other cooperative transcription factors, leading to activated gene expression of downstream target genes. In this regard, our current study indicated that deletion of the SP1 zinc-finger domain disrupts the interaction between SP1 and TEAD4. Notably, SP1 contains a C-terminal multimerization domain (D domain) that facilitates the formation of SP1 tetramers and the superactivation of promoters and/or enhancers with multiple adjacent SP1 binding sites [51]. Multimerization can expose various regions of DNA-bound or unbound SP1 for protein–protein interactions, which might explain our observation that deletion of the D domain disrupted the interaction of SP1 with TEAD4.

The serum is a strong stimulator of YAP/TEAD activity [38]. Moreover, PKCζ is activated by PI3K/PDK1 signaling in response to serum stimulation [52]. Here, we found that serum-stimulated PKCζ further enhanced the interaction of SP1 with TEAD4, which is in agreement with the activation of YAP/TEAD transcriptional activity upon serum stimulation to sustain tumor cell proliferation and survival. While previous studies have shown that PKCζ promotes cell survival via activation of NF-κB and acts as a pro-tumorigenic gene in pancreatic cancer and prostate cancer cells, more recent studies have demonstrated that PKCζ acts as a versatile tumor suppressor, especially in the intestine [53–56]. This discrepancy could be due to the diverse upstream signals that activate PKCζ and the different downstream substrates of PKCζ in different cellular contexts. In addition to its effect on serum, PKCζ can be activated by nutrient deprivation in response to stress and prevents metabolic reprogramming and activation of YAP/β-catenin under nutrient stress, thus leading to decreased CRC cell survival [54, 55]. Interestingly, loss of PKCζ induces nuclear localization of YAP/β-catenin in CRC cells under nutrient stress conditions but not under normal conditions [55]. In addition, PKCζ-KO MEFs grow more slowly than WT MEFs under normal conditions with enough nutrients; however, outgrowth of PKCζ-KO cells has been observed under serum- and nutrient-limiting conditions [57]. We speculate that PKCζ may play opposite roles in CRC cell proliferation under serum/nutrient-sufficient or nutrient-limiting conditions. Thus, although inhibiting PKCζ represents an indirect strategy to interfere with the SP1/TEAD4 interaction and inhibit the oncogenic activity of YAP/TEAD, it may be infeasible to use PKCζ inhibitors for the treatment of YAP-active CRC. In this study, we used mithramycin A (MMA), an FDA-approved chemotherapeutic agent that competitively blocks the binding of SP1 to gene promoters, to demonstrate that combination treatment with an SP1 inhibitor and a YAP/TEAD inhibitor could improve antitumor activity in CRC.

Transcriptional regulation of PDL1 and certain cytokines by YAP/TAZ creates an immunosuppressive TME and allows tumor cells to evade immune surveillance [13–15]. The YAP/TAZ-PDL1 regulatory axis has been detected in several cancers, including breast and lung cancers and melanoma [13–15]. However, we found that YAP/TAZ did not modulate PDL1 expression in CRC cells, indicating a context-dependent manner of transcriptional regulation of PDL1 by YAP/TAZ. Notably, YAP/TAZ’s regulation of PD-L1 transcription is human-specific, and TAZ does not regulate PDL1 expression or promoter activity in murine cell lines [14]. Thus, the biological relevance of the YAP/TAZ-PDL1 axis has not been explored in mouse models. Our discovery of the YAP/TAZ/SP1-VISTA regulatory axis in CRC is consistent with the immune evasion function of YAP/TAZ in human cancers. However, since VISTA is not expressed in the widely used CT26 and MC38 murine CRC cell lines, the biological significance of the YAP/TAZ-VISTA axis in vivo was not explored in this study. Furthermore, activation of YAP/TEAD frequently occurs during the administration of chemotherapy, radiation therapy, or targeted therapies for various cancers [6]. For example, a recent study revealed that anti-PD1 therapy activates YAP activity through IFN-γ-induced tumor cell YAP phase separation, resulting in adaptive resistance to anti-PD1 therapy [58]. We speculate that activation of YAP might account for the upregulation of VISTA during anti-CTLA4 therapy and anti-PD1 therapy [21, 22]. According to the CCLE database, VISTA is widely expressed in epithelial cancer cell lines. Notably, VISTA was recently reported to be highly expressed in malignant pleural mesothelioma (MPM) [59]. Given that mesothelioma is driven by the activation of YAP, it is likely that YAP/TEAD modulates VISTA expression in MPM [60]. Future studies investigating the biochemical and biological nature of the YAP/TAZ-VISTA axis in other types of cancer cells will provide insight regarding the underlying mechanisms of intrinsic or acquired resistance to immune checkpoint inhibitors (ICIs).

More than 10 mAb-based VSITA therapies are in the stage of preclinical and clinical development [17]. Reliable and accurate determination of VISTA expression is the basis of the application of these anti-VISTA therapy. Immunohistochemical analysis is a common method for assessing protein expression levels, especially in the clinic. Early studies of VISTA expression in CRC and lung cancer performed by using a noncommercial monoclonal antibody showed no VISTA expression in tumor cells [45]. However, evidences of tumor cell-specific expression of VISTA are emerging [25–27]. According to a recent report of VISTA expression in CRC cohorts comprising 816 patients, 66% of the patient samples were VISTA-positive in the stroma, while only 1.8% of the tumor tissues were VISTA-positive [25]. In contrast, RNA-seq data from CCLE indicated that VISTA is highly expressed in CRC cell lines, which is consistent with our western blot analysis here showing expression of VISTA in more than 10 CRC cell lines. Also, VISTA expression in CRC cells is supported by the single-cell sequencing of CRC tissue [43]. Consistent with the public scRNA-seq data of CRC, FACS analysis in this study revealed that tumor cell-specific expression of VISTA was positive in all CRC tissues, with VISTA-positive CRC cells ranging from 15.7 to 36.8%, suggesting that VISTA is indeed expressed in tumor cells. This discrepancy could be due to the different VISTA antibodies used in different studies. Our study here suggested that this discrepancy could also be due to the glycosylation of VISTA. A protein deglycosylation strategy was previously used to facilitate the structure determination of VISTA protein [61]. We reasoned that glycosylation of VISTA could render its polypeptide antigens inaccessible to VISTA antibodies. Indeed, our study demonstrated that removing glycosylation can enhance the anti-VISTA signal in IHC assays, especially in tumor cells, probably implying the higher VISTA glycosylation level in tumor cells. Contrasting to a previous study reporting that VISTA expression is correlated with good prognosis in CRC patients, our study showed that enhanced anti-VISTA signaling after deglycosylation is a poor prognostic marker for CRC patients. In this regard, further studies are required to fully address the prognostic value of VISTA.

The recent discovery of cancer cell-intrinsic PD-L1 signals indicates the important roles of PD-L1 in regulating tumor growth and survival pathways, stemness, DNA damage responses and gene transcription [62]. Since VISTA can function as both a ligand and a receptor, cancer cells expressing VISTA might also exert important biological effects on tumor cells [16–18]. V-set and Ig domain-containing 3 (VSIG3) is a ligand that interacts with VISTA at physiological pH [19]. VSIG3 is highly expressed in gastrointestinal tumors, including CRC [19]. Tumor cell-derived VSIG3 can interact with VISTA expressed on T cells, which results in the inhibition of T-cell function [19]. Given our discovery of universal VISTA expression in CRC cells, the VSIG3-VISTA interaction might mediate cross-talk between CRC cells, an intriguing issue that warrants further investigation. The limitations of this study include the lack of in vivo mouse model to explore the functional relevance of YAP/TAZ/SP1-VISTA regulatory axis in immune evasion in CRC. The mouse xenograft model in immunocompetent mouse is not feasible due to the absence of endogenous VISTA expression in the widely used CRC mouse cell lines. The humanized mice or YAP/VISTA transgenic/gene knockout genetic mouse models could be used to overcome the technical issue in this study. Nevertheless, YAP/TEAD4/SP1-induced VISTA expression in tumor cells provides a new strategy for targeting VISTA for immune checkpoint therapy.

## Materials and methods

### Cell lines, cell culture and transfection

All cancer cell lines (HCT116, SW480, LoVo, Caco2, HT29, RKO, DLD1, HCT15, SW620, A549, and DU145) and HEK293T cells were purchased from American Type Culture Collection (ATCC) and maintained in DMEM or RPMI-1640 medium (HyClone) supplemented with 10% fetal bovine serum (BI), 100 units/ml penicillin and 100 μg/ml streptomycin (Sangon Biotech) in a 37 °C incubator with 5% CO^2^. Transient transfections with plasmid DNA or siRNA were performed using polyethylenimine (PEI; Polysciences) and Lipofectamine RNAiMax (Invitrogen), respectively, following the manufacturer’s protocols. The siRNA sequences are listed in Supplementary Table 1.

### RNA isolation and qRT‒PCR

Total RNA was extracted from the indicated cells using TRIzol reagent (Invitrogen) following the manufacturer’s protocols. cDNA was synthesized using HiScript III RT SuperMix (Vazyme). Quantitative RT–PCR (qRT–PCR) was performed using qPCR SYBR Green Master Mix (Vazyme) on an ABI 7500 PCR System (Applied Biosystems). The sequences of primers used are listed in Supplementary Table 2.

### Western blot analysis and immunoprecipitation

Western blot analysis and immunoprecipitation were performed as previously described [63]. For endogenous immunoprecipitation, the cell lysates were incubated with the indicated primary antibodies at 4 °C overnight. Then, protein A/G agarose beads were added, and the lysates were incubated for an additional 2 h. The beads were washed three times with lysis buffer, resuspended in loading buffer and boiled at 95 °C for 10 min before being subjected to immunoblotting. The primary antibodies used in this study are listed in Supplementary Table 3.

### Duolink proximity ligation assay

A proximity ligation assay (PLA) was performed by using a Duolink In Situ Detection Kit (Sigma) according to the manufacturer’s protocol. Briefly, HCT116 cells were seeded on coverslips, fixed in 4% PFA for 30 min, permeabilized in 0.5% Triton X-100 for 10 min, washed 2 times with PBS, and blocked with blocking reagents for 1 h at room temperature. After blocking, the cells were incubated with primary antibodies against SP1 (1:100, Rb, Abclonal, A19649), YAP (1:100, Ms, Santa Cruz Biotechnology, sc-101199), TAZ (1:100, Ms, BD Bioscience, #560235) and TEAD4 (1:100, Ms, Santa Cruz Biotechnology, sc-390578) for 1 h. After PLA probe incubation, ligation, and amplification, the cells were photographed, and the number of PLA foci was quantified.

### Luciferase reporter assay

The VISTA promoter (–2000/ + 100 bp) was cloned and inserted into the pGL3-Basic reporter vector. VISTA reporters with mutations in SP1 and/or TEAD binding motifs were generated by using a KOD Plus Mutagenesis Kit (TOYOBO) according to the manufacturer’s protocol. To assess the luciferase activity of the VISTA reporters, HEK293T cells were plated in 24-well plates and cotransfected with the VISTA luciferase reporter, CMV-Renilla luciferase reporter and other indicated plasmids. Luciferase activity was measured 48 h after transfection by using the Dual Luciferase Reporter Assay System (Promega). The relative luciferase activity was determined by normalizing the firefly luciferase activity to the Renilla luciferase activity.

### Tissue microarray and immunohistochemistry

All human tissues were obtained in the Department of Colorectal and Anal Surgery, Xinhua Hospital, Shanghai Jiao Tong University School of Medicine, from January 2008 to December 2018, according to the inclusion and exclusion criteria. This study was approved by the ethics committee of Xinhua Hospital. Institutional review board approval and informed consent were obtained for all sample collections. A total of 190 paired CRC and normal mucosa specimens were included and used to prepare a tissue microarray for immunohistochemistry (IHC) analysis. The details of the primary antibodies used in this study are presented in Supplementary Table 3.

### Cell proliferation and migration assays

Cell Counting Kit-8 (CCK-8) and colony formation assays were performed to evaluate cell proliferation. Cell migration was measured by Transwell assays. For drug combination treatment, cells were seeded into 96-well plates (3000–5000 cells/per well) and cultured with the indicated concentrations of drugs for 48 h. Then, cell viability was measured by CCK-8 assays, and the combination effects were analyzed by the Chou–Talalay combination index (IC) method [64]. CI values < 1, =1, and >1 indicate synergistic, additive, and antagonistic effects, respectively, between the drugs. For the colony formation assay with drug combination treatment, cells were seeded into 6-well plates (5000–10,000 cells/per well) and cultured with the indicated concentration of drugs for 10 days. The colonies were fixed and stained with crystal violet, and the number of colonies was counted for statistical analysis.

### Xenograft tumor models

All animal procedures were approved by the animal care and use committees of Xinhua Hospital. Male nude mice (6-8 weeks old) were used to construct the xenograft tumor models were as previously described. According to the criteria of the Animal Care and Use Committee of Xinhua Hospital, the maximal tumor burden permitted was less than 10% body weight; none of the mice exceed the maximal tumor burden at any point during the experiment. For the in vivo drug combination treatment, when the tumor volume reached approximately 100 mm^3^, the mice were randomly assigned to the indicated groups and treated with vehicle, 0.1 mg/kg MMA (i.p., every three days), 25 mg/kg VP (i.p., every other day), 10 mg/kg VT107 (i.p., every other day) or a combination of MMA and VP/VT107 (*n* = 6 mice per group). Tumor volumes were calculated with the following formula: 0.5 × (largest diameter) × (smallest diameter)^2^. At the experimental endpoint, the mice were sacrificed, and the tumors were harvested, imaged and weighed.

### Intestinal organoid culture and organoid viability assay

Patient-derived organoid (PDO) culture and organoid viability assays were performed in accordance with previously studies [65]. Institutional review board approval and informed consent were obtained for all CRC samples used for establishing the CRC PDOs. CRC PDOs were seeded in 96-well plates. After 48 h, the organoids were treated with the indicated concentrations of MMA (MCE) and/or VP (Selleck) for 48 h. The viability was quantified using CellTiter-Glo (Promega), and the relative viability was normalized to that in the DMSO group.

### Isolation of CD8+ T cells and coculture with tumor cells

Human peripheral blood was collected from healthy volunteers recruited from Xinhua Hospital, Shanghai Jiao Tong University School of Medicine. Institutional review board approval and informed consent were obtained from all subjects. This study was approved by the ethics committee of Xinhua Hospital. Peripheral blood mononuclear cells (PBMCs) were obtained from whole blood with Ficoll-Paque PLUS (GE Healthcare). CD8^+^ T cells were isolated from PBMCs using a human CD8 + T-Cell Isolation Kit (Miltenyi Biotec) and stimulated with 2 mg/ml anti-CD3 antibody and 1 mg/ml anti-CD28 antibody (eBioscience).

For the T-cell coculture assay, 10000 targeted CRC cells were plated in 96-well plates and then cocultured with activated CD8^+^ T cells at a 1:10 ratio for 48 h. After coculture, the CRC cells and CD8^+^ T cells were harvested to examine cell apoptosis and cytokine production by flow cytometry, respectively.

### Flow cytometry

For apoptosis analysis, a FITC Annexin V Apoptosis Detection Kit I (BD Biosciences) was used to analyze the apoptosis of the cocultured CRC cells according to the manufacturer’s protocol. For intracellular cytokine staining, CD8 + T cells were treated with Cell Stimulation Cocktail (plus protein transport inhibitors) (eBioscience) for 4 h. Then, the cells were fixed and permeabilized using a Fixation/Permeablization Kit (BD Biosciences) and stained with antibodies against intracellular cytokines and granzyme B. For human CRC samples, tumor tissues were digested into single-cell suspensions by digestion media (0.1 mg/ml collagenase IA, 60 U/ml DNase I). An anti-CD326 antibody was used to identify epithelial cells in human CRC tissues, and a VISTA antibody was used to measure the expression of VISTA in epithelial cells. The detailed antibody information is provided in Supplementary Table 3. All flow cytometric analyses were performed with a FACSCanto II instrument (BD Biosciences) and FlowJo software (TreeStar).

### Deglycosylation assay of cell lysates

The cells were lysed in lysis buffer (20 mM Tris-HCl (pH 8.0), 150 mM NaCl and 1% NP40) supplemented with protease inhibitor cocktails (Roche) for 30 min at 4 °C. Cell lysates were deglycosylated by treatment with PNGase F (New England Biolabs) according to the manufacturer’s directions. Briefly, cell lysates were denatured with glycoprotein denaturing buffer at 100 °C for 10 min. Denatured cell lysates were chilled on ice and then incubated with PNGase F at 37 °C for 1 h. Deglycosylated proteins were analyzed by western blotting.

### Immunofluorescence staining assay with deglycosylation

Cells were seeded on coverslips and then fixed in 4% PFA for 30 min at room temperature. Fixed cells were denatured with glycoprotein denaturing buffer at 100 °C for 10 min. After denaturation, the cells were treated with or without 5% PNGase F at 37 °C overnight and then subjected to immunofluorescence staining. In brief, the cells were permeabilized in 0.5% Triton X-100 for 10 min, washed 3 times with PBS and blocked with 5% bovine serum albumin (BSA; Sigma) for 1 h at room temperature. After blocking, the cells were incubated with primary antibodies against VISTA (Cell Signaling Technology, #54979) at 4 °C overnight. FITC-conjugated secondary antibody was used at room temperature for 1 h, and the nucleus was stained with DAPI (Sigma) for 30 min. Images were obtained with an Olympus IX81.

### IHC assay with deglycosylation

Deglycosylation of formalin-fixed paraffin-embedded (FFPE) tissue sections by PNGase F was performed as described by other groups [46]. After deparaffinization and antigen retrieval, the tissue sections were denatured with glycoprotein denaturing buffer for 3 h at room temperature. The denatured sections were treated without or with 5% PNGase F at 37 °C overnight and then subjected to IHC staining as described above. The histoscore (H-score) was calculated to evaluate the IHC results following a previously described procedure [46]. The semiquantitative score was calculated by multiplying the staining intensity (0, negative; 1, weak; 2, intermediate; 3, strong) and percentage of positive staining (resulting in a total score ranging from 0-300). Two histopathologists were assigned to review the slides and score the staining in a blinded manner.

### ChIP‒qPCR and ChIP‒seq analysis

Chromatin immunoprecipitation (ChIP) was performed on HCT116 cells with a Magna ChIP Kit (Merck, 17-610). Briefly, the cells were cross-linked with 1% formaldehyde for 10 min, after which the reaction was stopped with 0.125 M glycine for 5 min at room temperature. After washing, lysis and sonication, the chromatin fraction was incubated with anti-SP1 (ABclonal, A19649), anti-YAP (Cell Signaling Technology, #14074) or control anti-IgG (Cell Signaling Technology, #3900) overnight at 4 °C. Chromatin-bound beads were subjected to extensive washing and elution. The eluted chromatin was decrosslinked, purified, and subsequently prepared for qPCR analysis. The qPCR primers used to amplify the YAP target genes and VISTA are provided in Supplementary Table 3. The ChIP-seq data for TEAD4 (ENCSR000BVJ) and SP1 (ENCSR000BSF) in HCT116 cells were downloaded from the ENCODE database. DeepTools2 was used to generate heatmaps, and Integrated Genomics Viewer (IGV, Broad Institute) was used to visualize the ChIP-seq peak data [66, 67].

### Statistics

All the statistical analyses were carried out with GraphPad Prism 8.0 and SPSS 22.0 software. Student’s t test and the nonparametric test were used to assess differences between two groups. One-way ANOVA with Dunnett’s multiple comparison test was used to assess the statistical significance for the experiments with >2 independent groups. For the CCK-8 and xenograft growth curve assays, two-way ANOVA with Dunnett’s multiple comparison test was performed to assess the statistical significance. Survival analysis was performed using Kaplan–Meier plots and the log-rank test. Pearson correlation analyses were used to evaluate the correlations between mRNA expression levels in the TCGA colorectal cancer database. All the data are presented as the means ± S.D. or means ± S.E.M. of at least three independent experiments unless otherwise noted in the figure legends. Differences were considered to be significant at *p* < 0.05 (*p < 0.05, **p < 0.01, ***p < 0.001).

## Supplementary information

Original western blots

Supplementary Figures and Tables

## Data Availability

The transcriptomic data generated by this study have been deposited in the NCBI’s Gene Expression Omnibus database (GSE179314). The data generated in this study are available upon request from the corresponding author.
