# Supplementary material for: YAP/TEAD4/SP1-induced VISTA expression as a tumor cell-intrinsic mechanism of immunosuppression in colorectal cancer
Source: Cell Death Differ. 2025 Jan 28;32(5):911–25. doi: 10.1038/s41418-025-01446-2 (PMC12089306; doi:10.1038/s41418-025-01446-2)

Figure 2

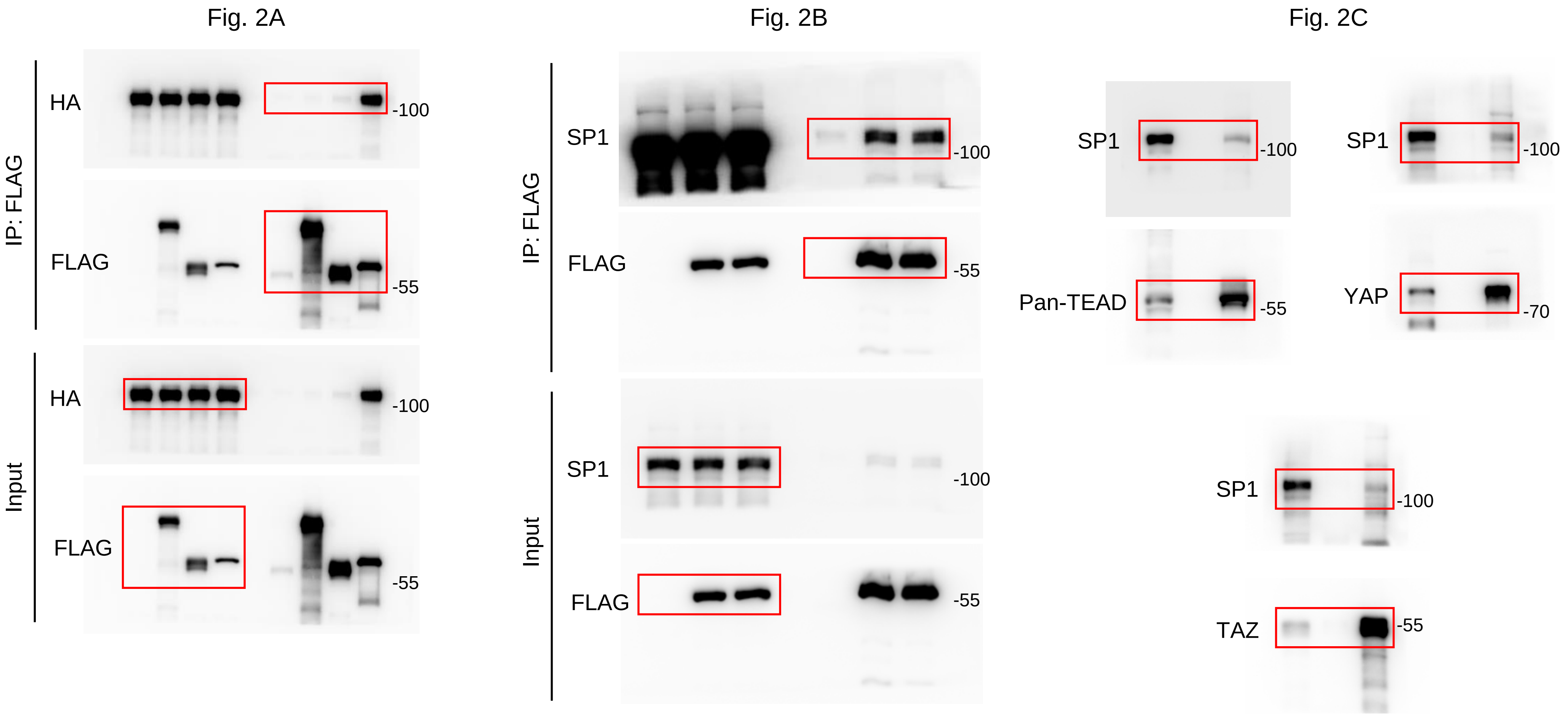

Fig. 2A
IP: FLAG
HA
-100
FLAG
-55
Input
HA
-100
FLAG
-55
Fig. 2B
IP: FLAG
SP1
-100
FLAG
-55
Input
SP1
-100
FLAG
-55
Fig. 2C
SP1
-100
Pan-TEAD
-55
SP1
-100
YAP
-70
SP1
-100
TAZ
-55

Figure 2

Fig. 2E

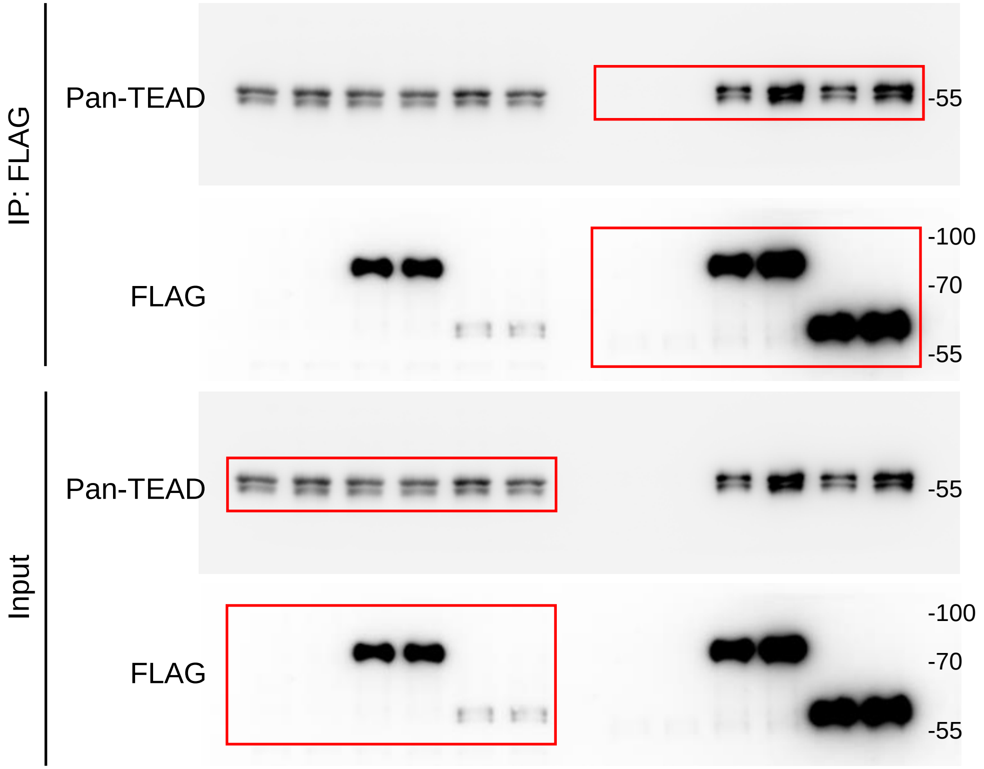

Fig. 2F

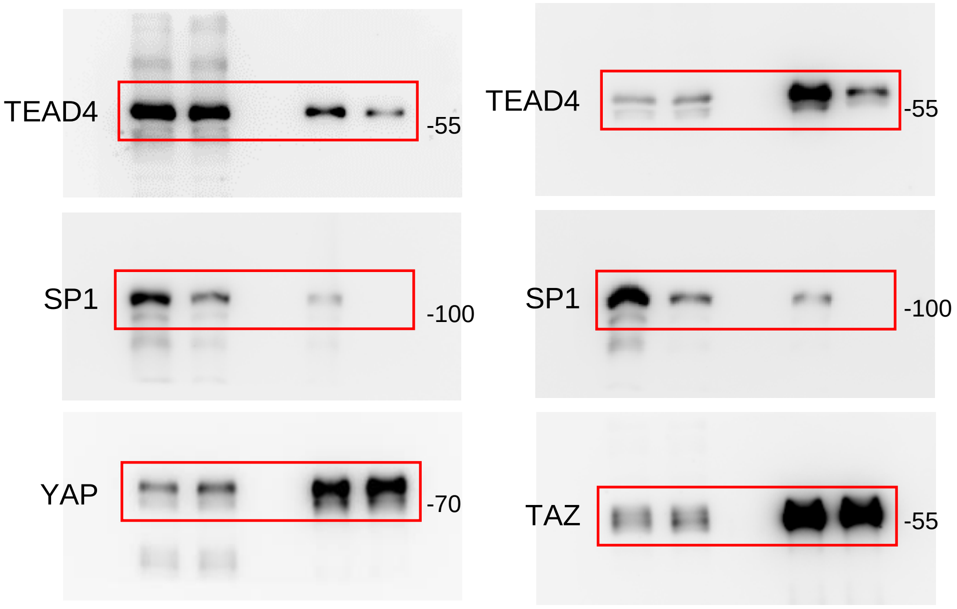

Figure 2

Fig. 2G

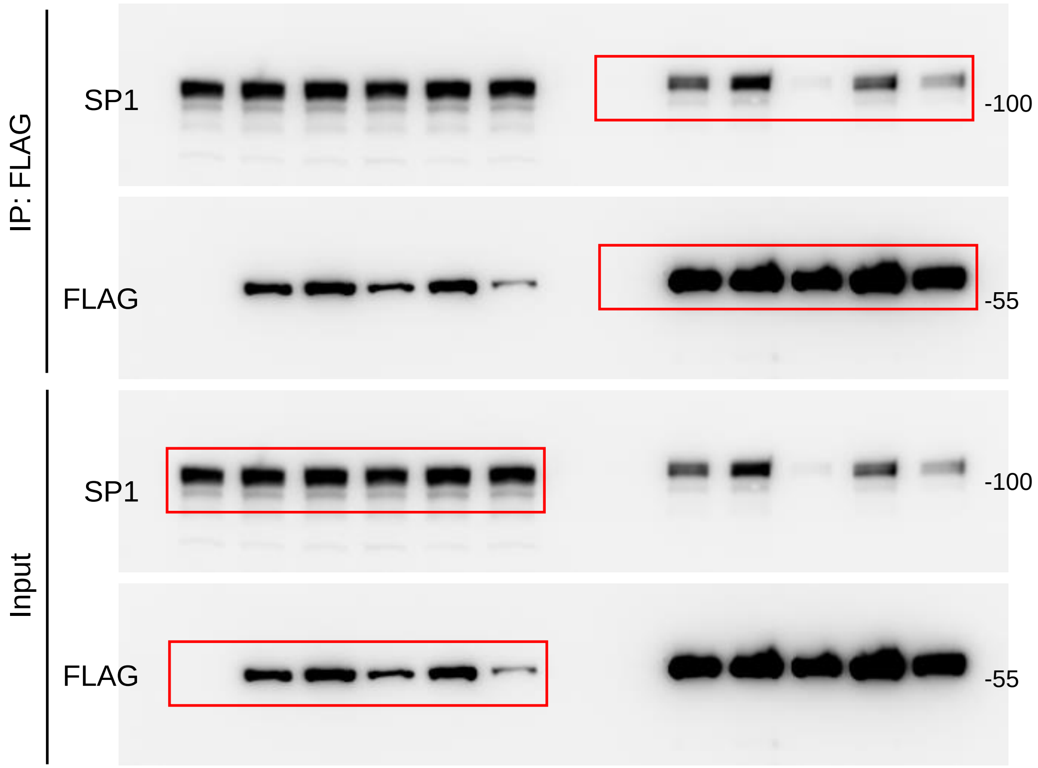

Fig. 2I

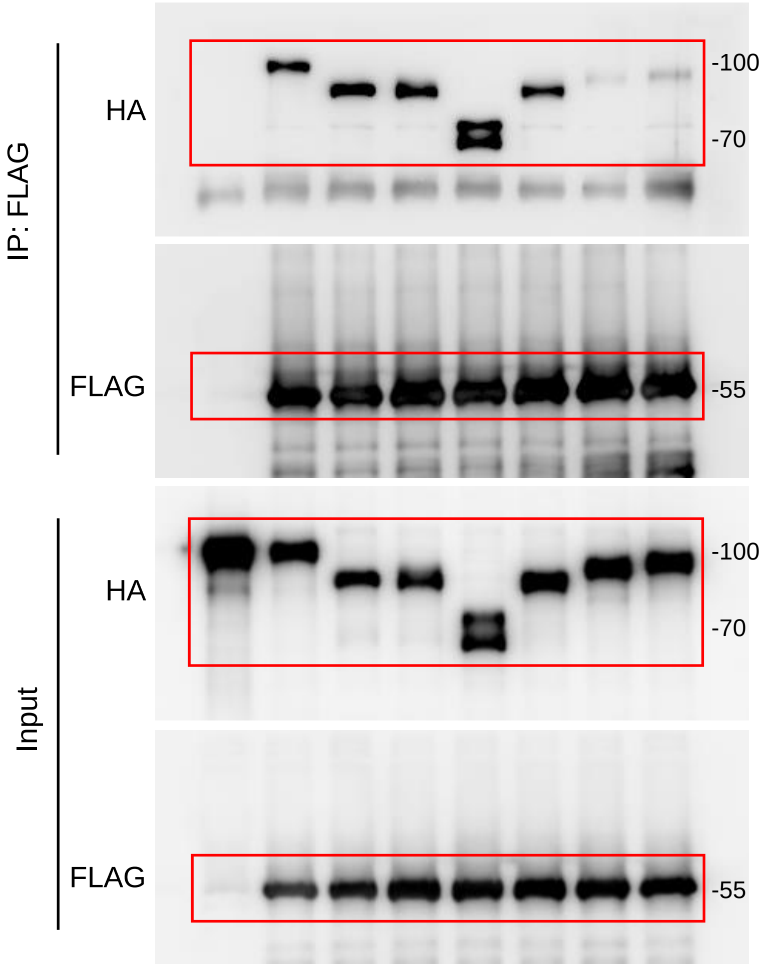

Figure 3

Fig. 3A

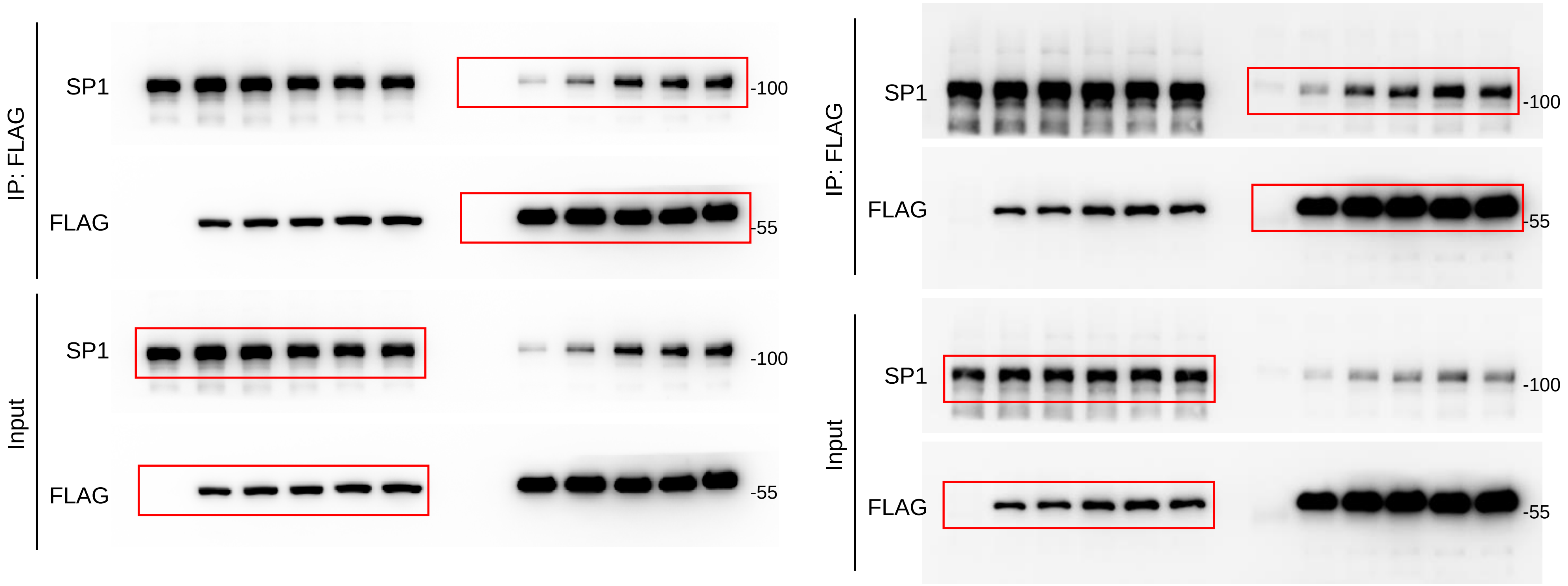

Figure 3

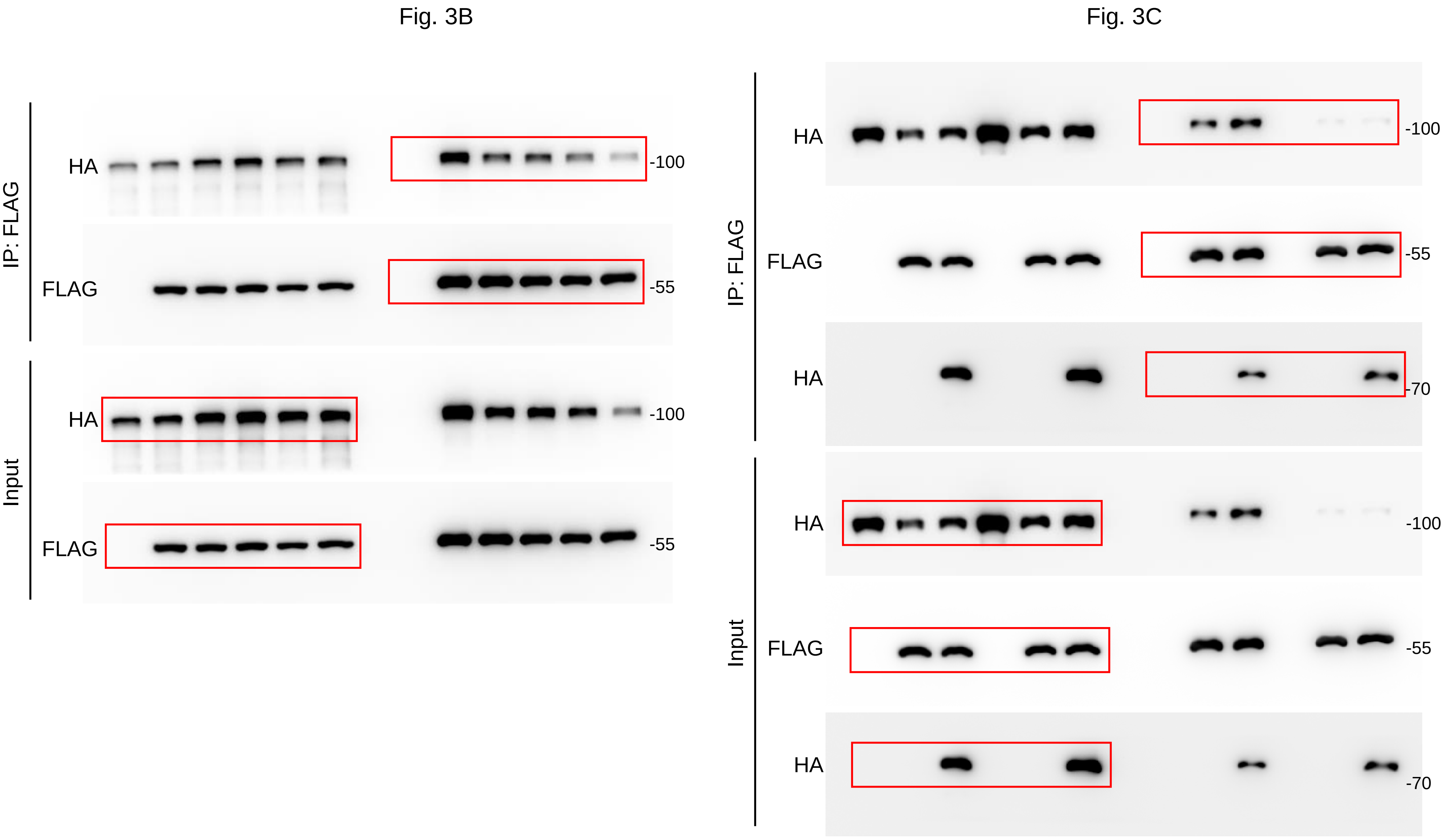

Figure 3

Fig. 3D

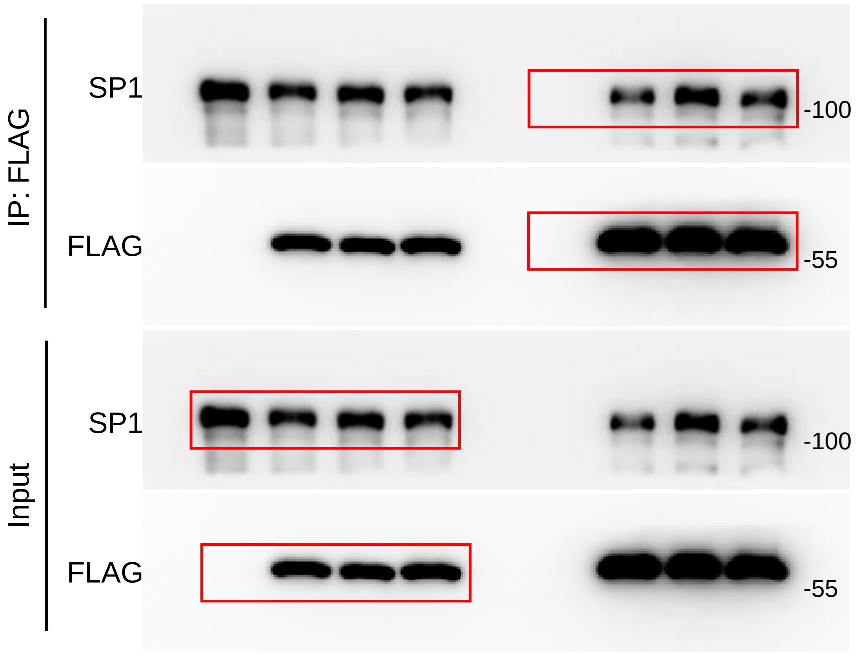

Fig. 3E

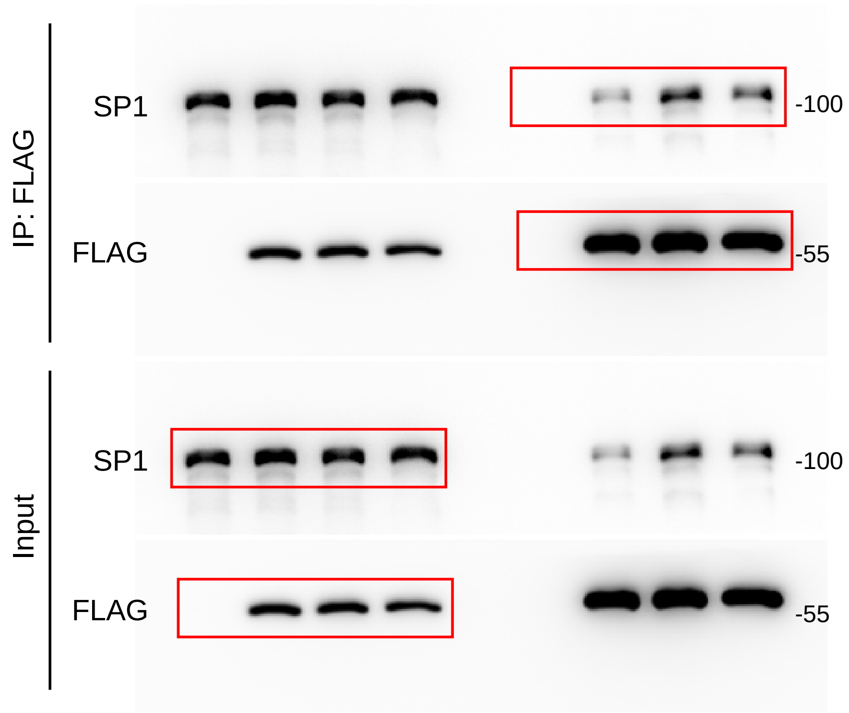

Figure 4

Fig. 4C

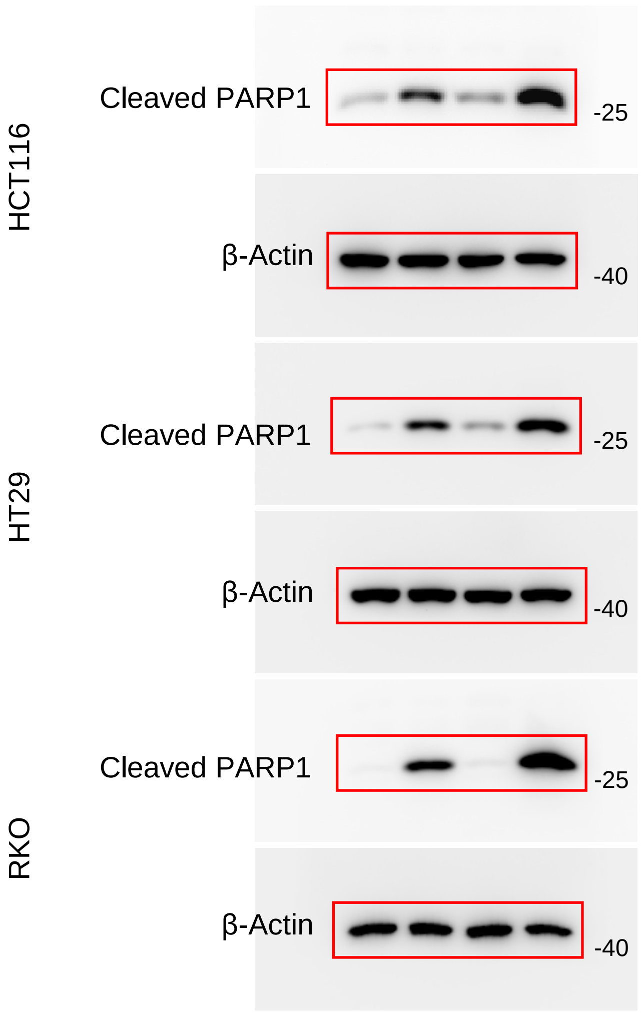

Figure 5

Fig. 5C

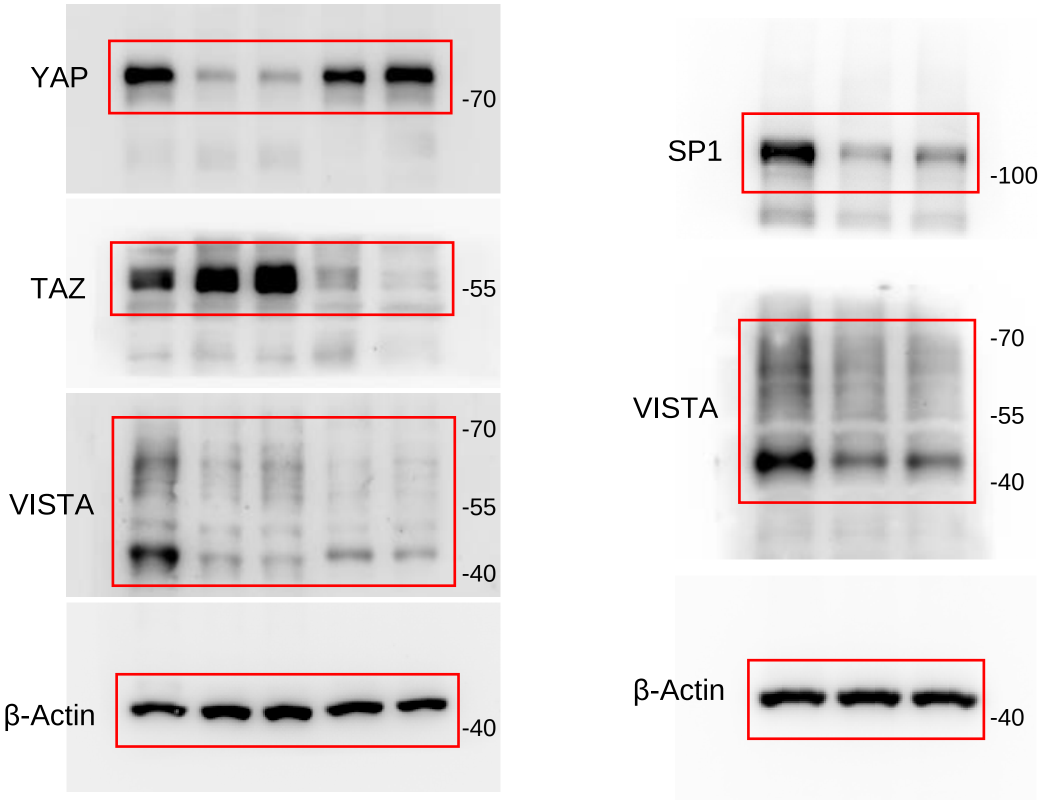

Figure 6

Fig. 6A

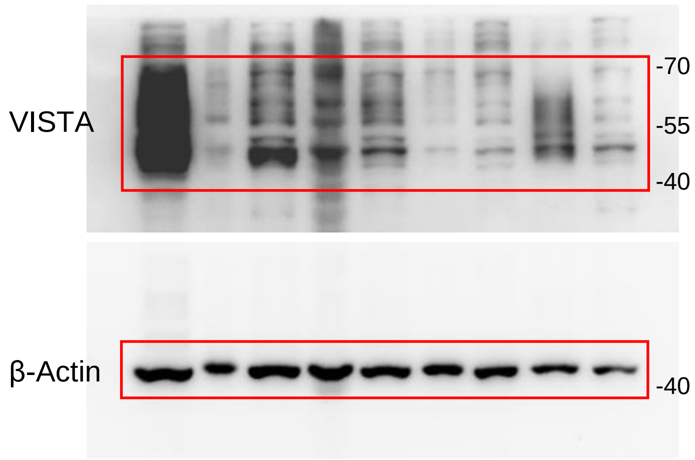

Fig. 6D

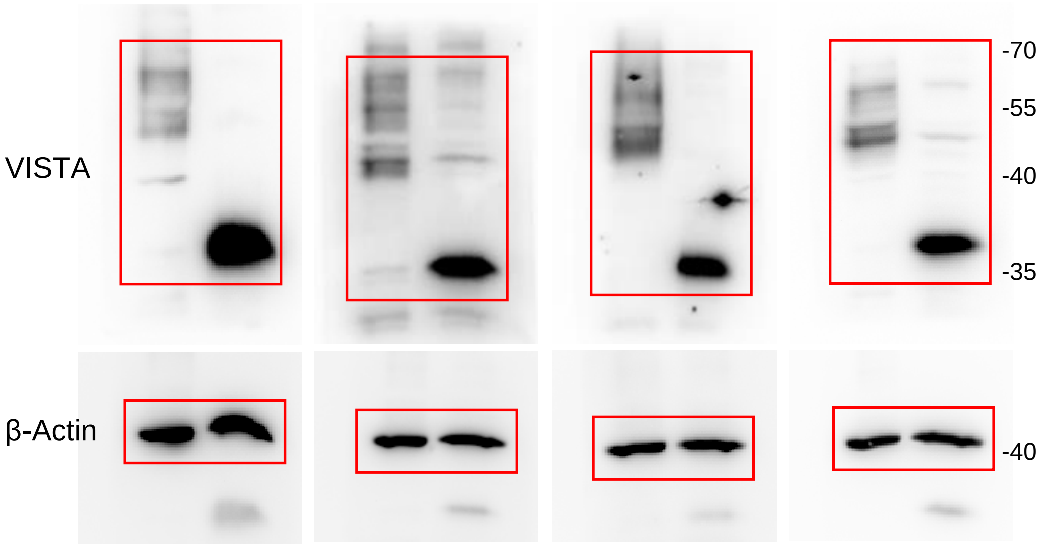

Figure S1

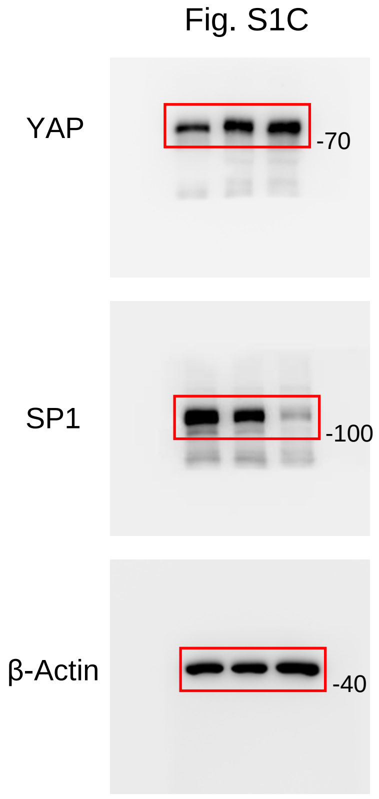

Figure S2

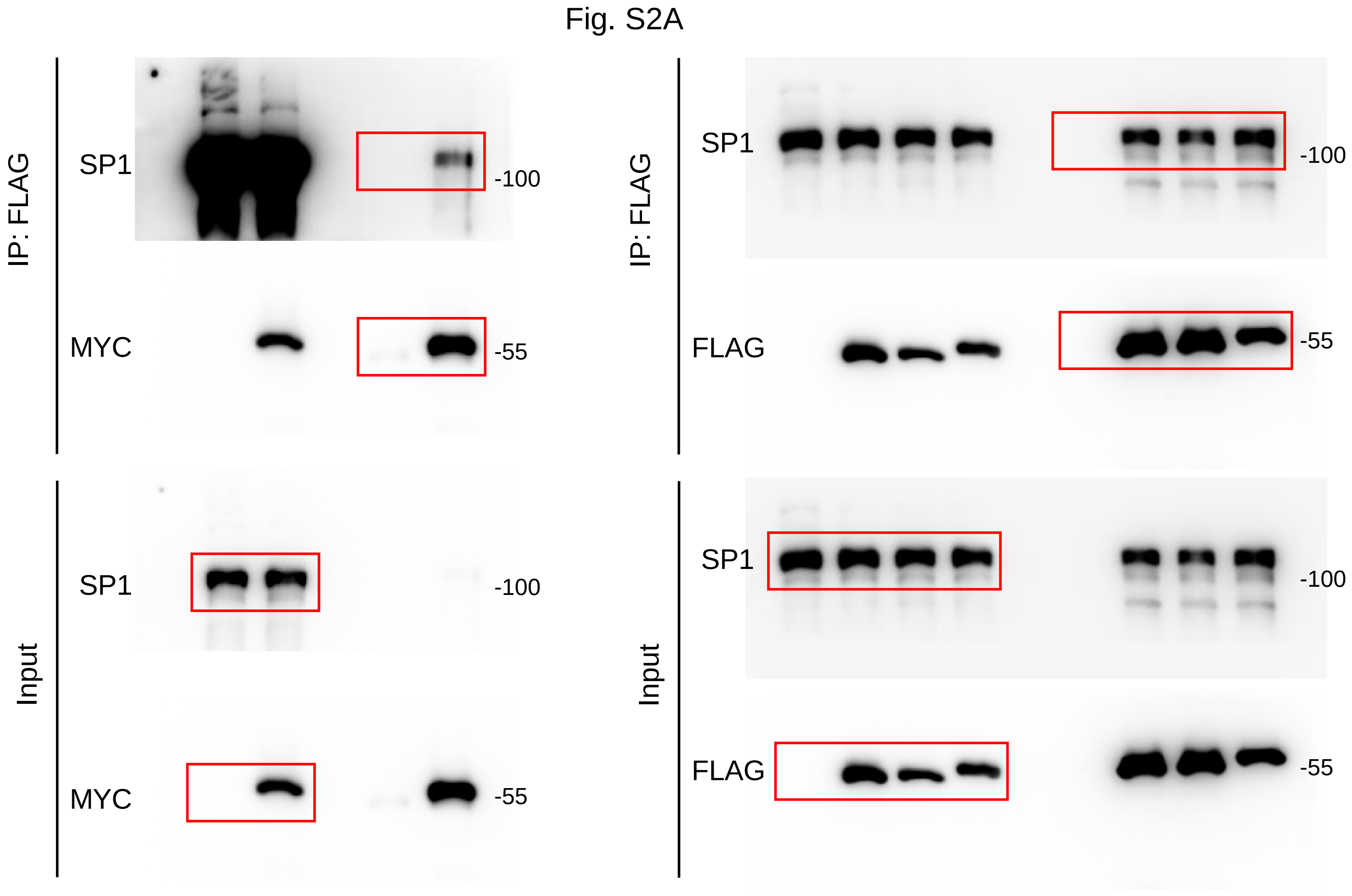

Figure S3

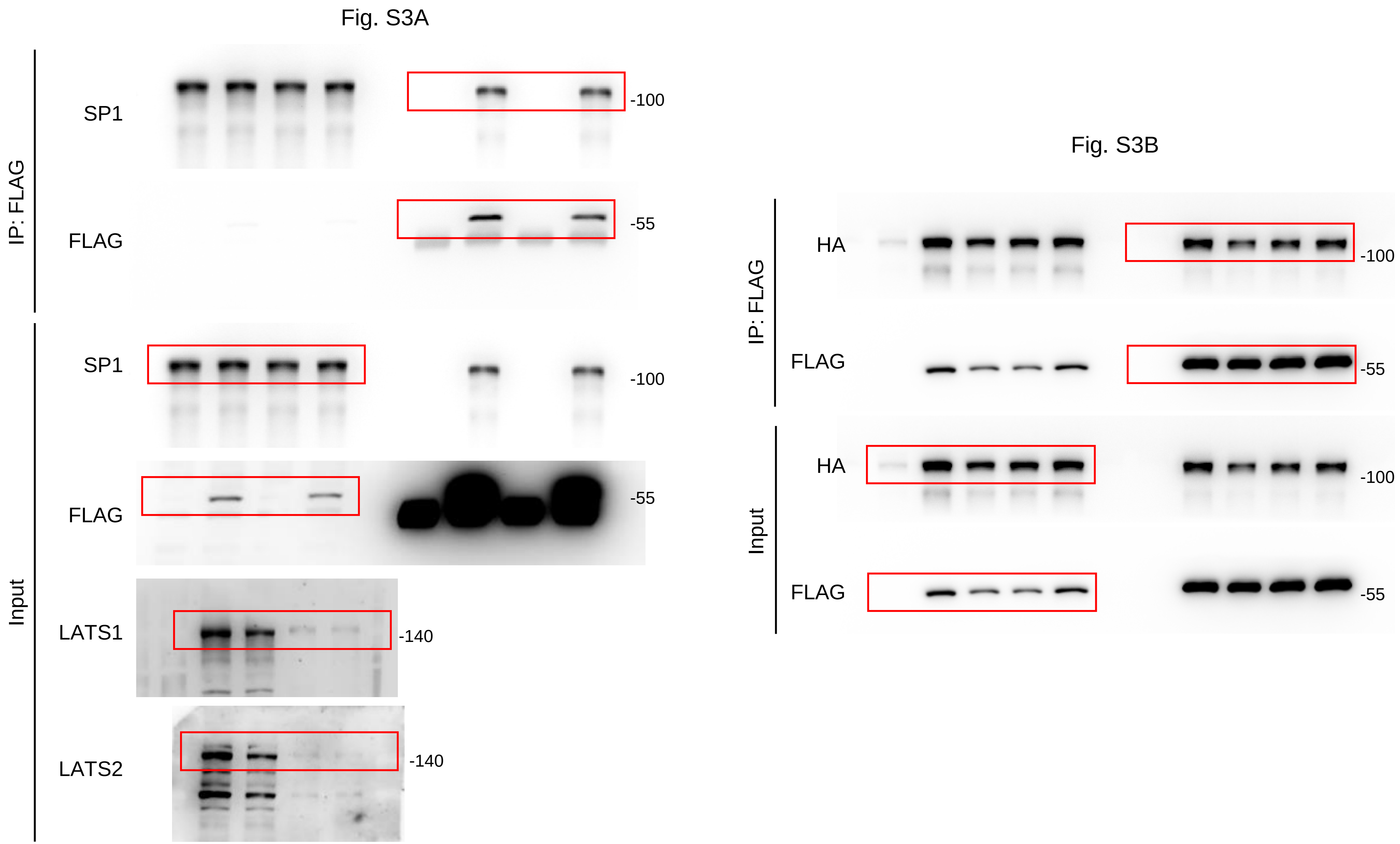
Fig. S3A
IP: FLAG
SP1
-100
FLAG
-55
Input
SP1
-100
FLAG
-55
LATS1
-140
LATS2
-140
Fig. S3B
IP: FLAG
HA
-100
FLAG
-55
Input
HA
-100
FLAG
-55

Figure S3

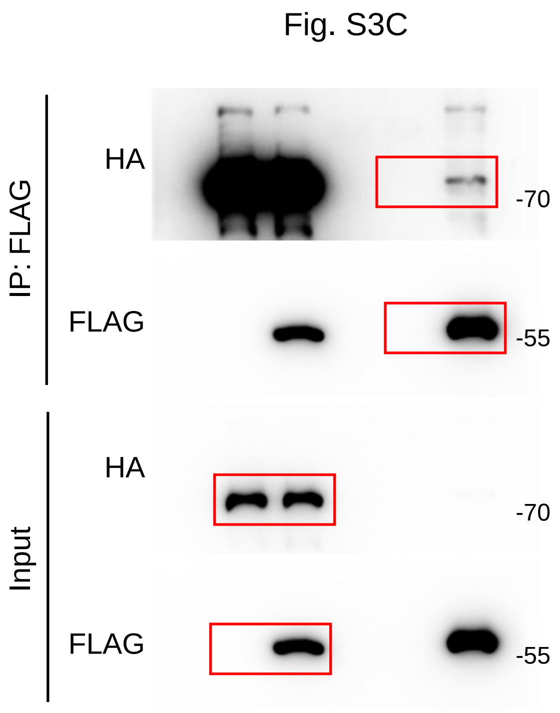

Figure S4

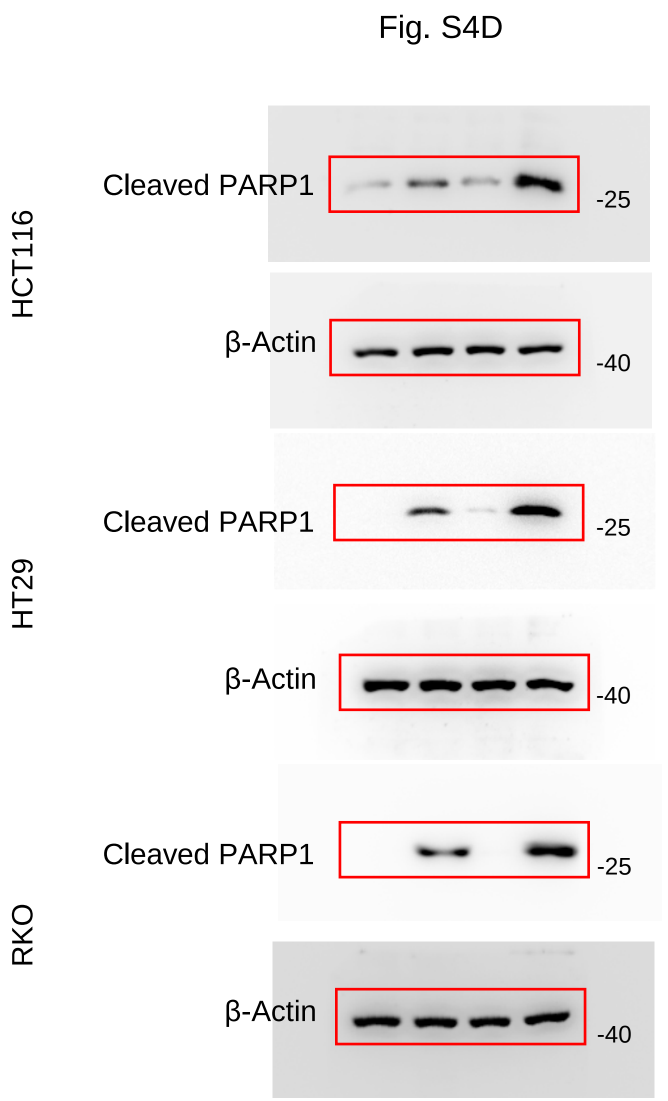

Figure S5

Fig. S5A

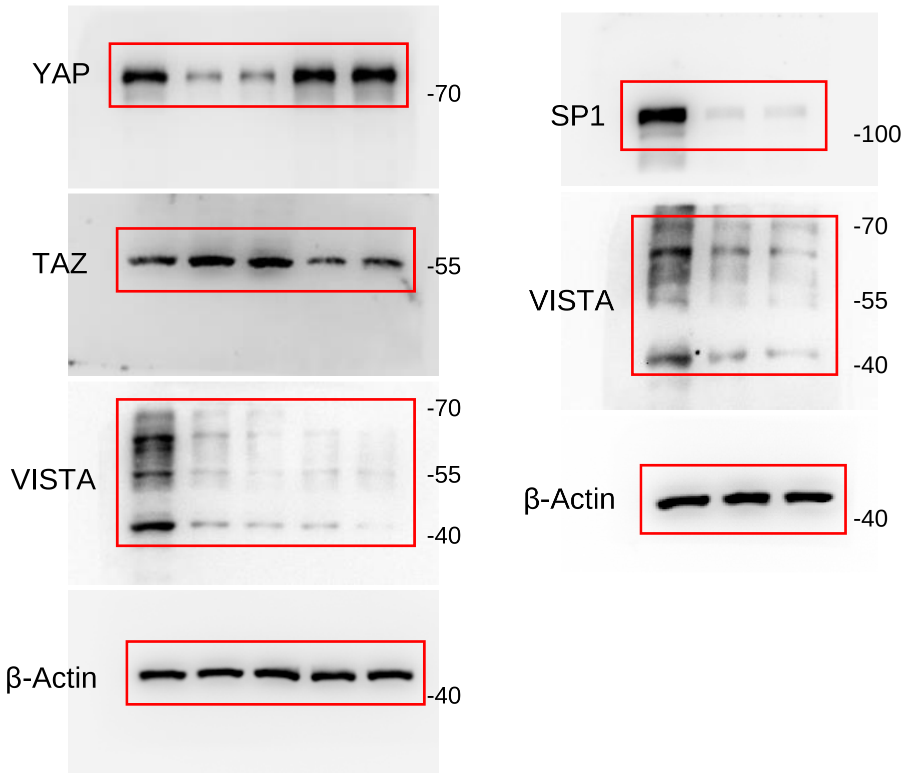

Fig. S5E

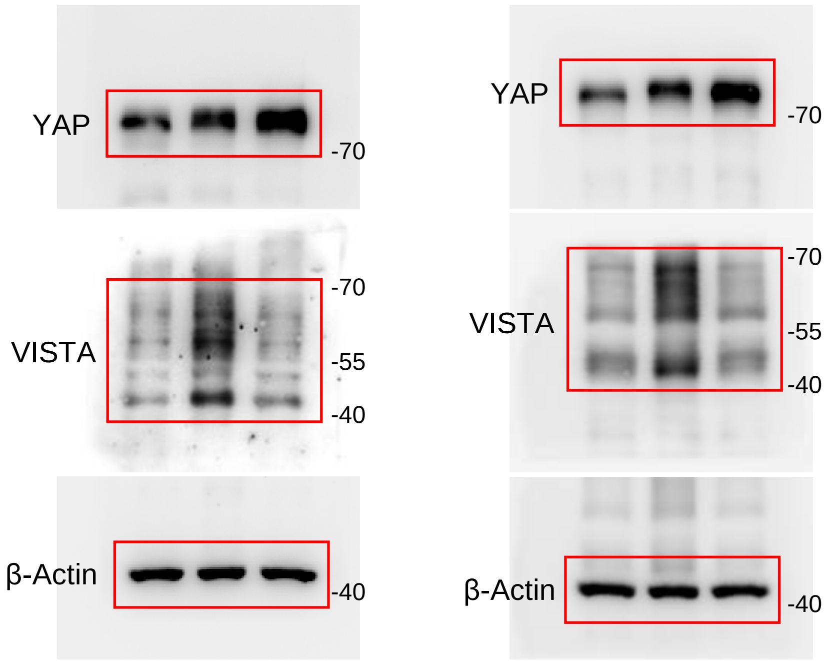

Figure S6

Fig. S6B

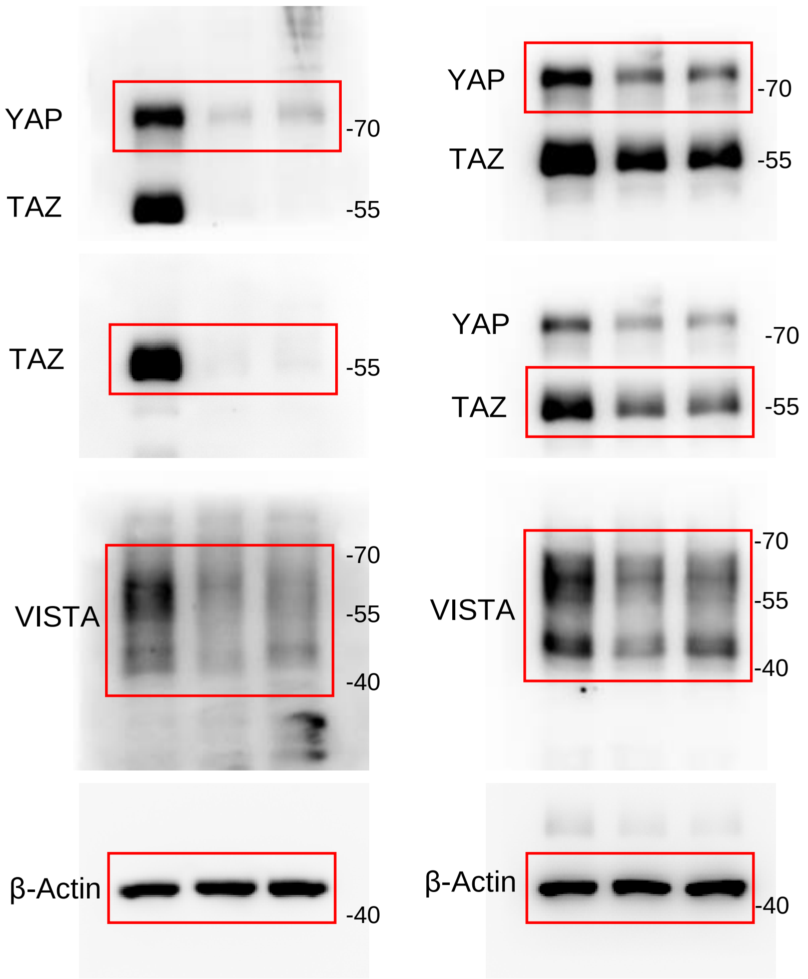

Fig. S6D

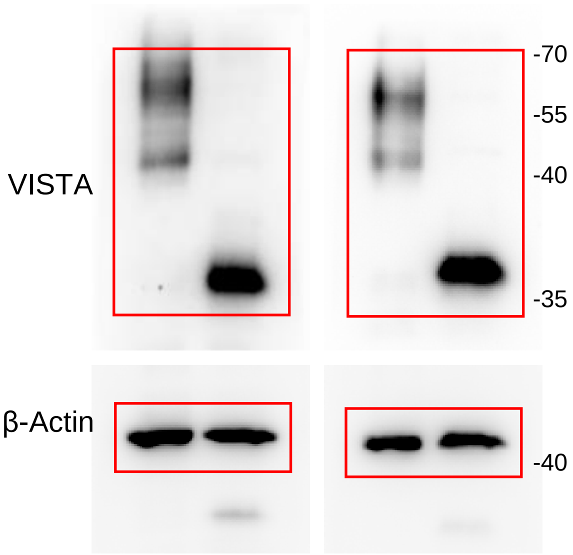

Fig. S6E

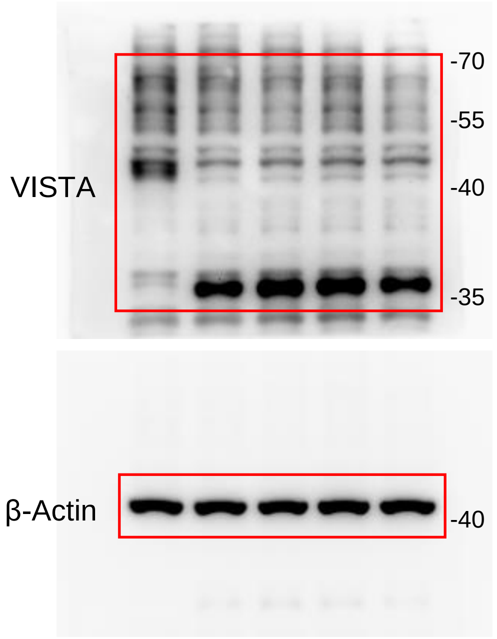

Supplement: Supplementary file 1 — Original western blots [file 41418_2025_1446_MOESM1_ESM.pdf]
