# Supplementary material for: YAP/TEAD4/SP1-induced VISTA expression as a tumor cell-intrinsic mechanism of immunosuppression in colorectal cancer
Source: Cell Death Differ. 2025 Jan 28;32(5):911–25. doi: 10.1038/s41418-025-01446-2 (PMC12089306; doi:10.1038/s41418-025-01446-2)
Supplement: Supplementary file 2 — Supplementary Figures and Tables [file 41418_2025_1446_MOESM2_ESM.docx]

**
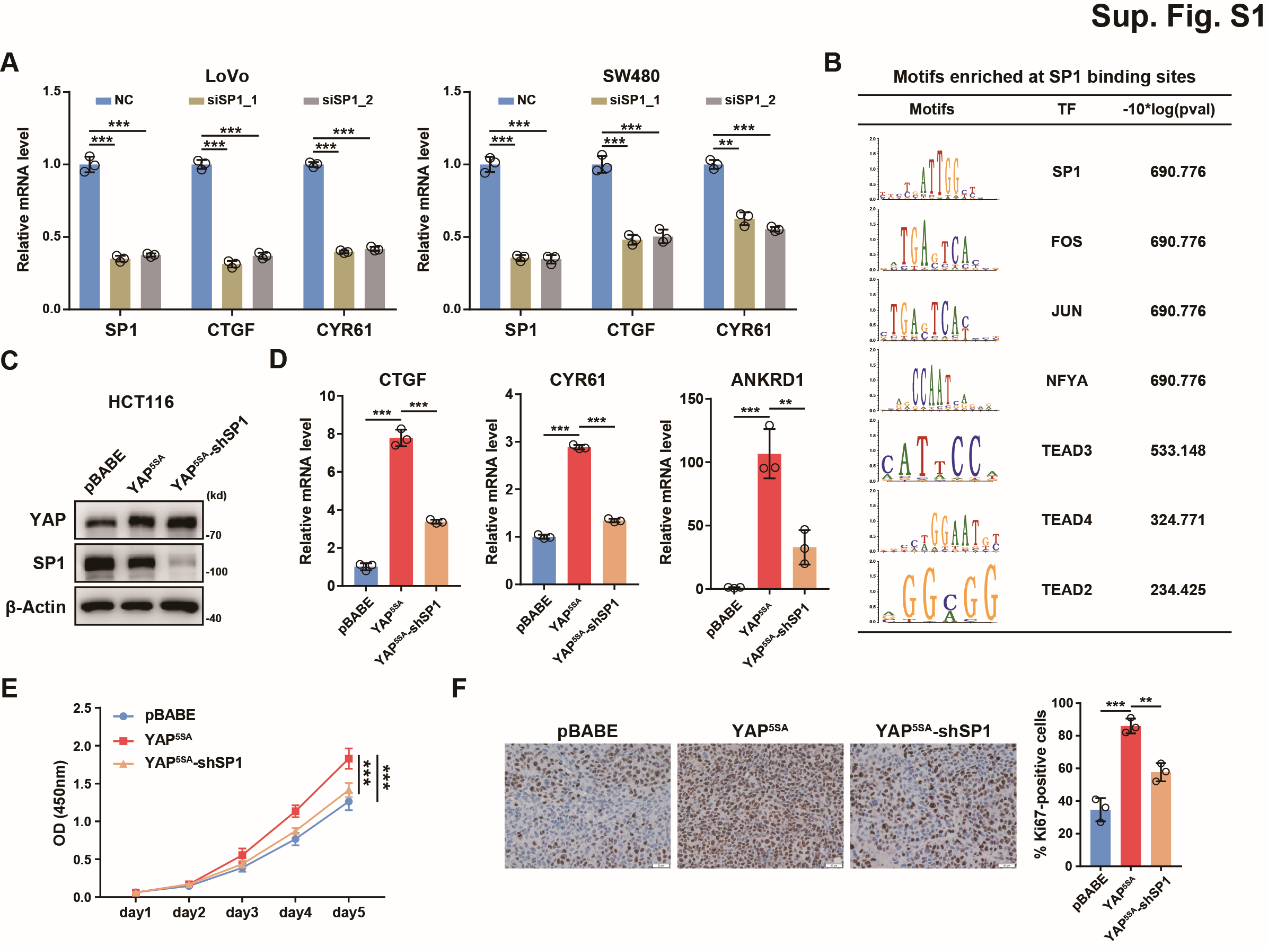
**

**Supplementary Figure S1. Related to Figure 1.**

1. qPCR analysis of the SP1, CTGF and CYR61 mRNA levels in LoVo and SW480 cells with SP1 knockdown.
2. Motif enrichment analysis of the SP1 binding sites in HCT116 cells. The data were extracted from the Cistrome database.
3. Generation of SP1-knockdown HCT116 cells overexpressing YAP^5SA^. Western blotting was performed to determine the expression of SP1 and YAP.
4. qPCR analysis of the CTGF, CYR61 and AKNRD1 mRNA levels in SP1-knockdown HCT116 cells overexpressing YAP^5SA^.
5. CCK8 assays of SP1-knockdown HCT116 cells overexpressing YAP^5SA^.
6. Representative images and quantification of IHC staining for Ki67 in xenograft tumors derived from SP1-knockdown HCT116 cells overexpressing YAP^5SA^.


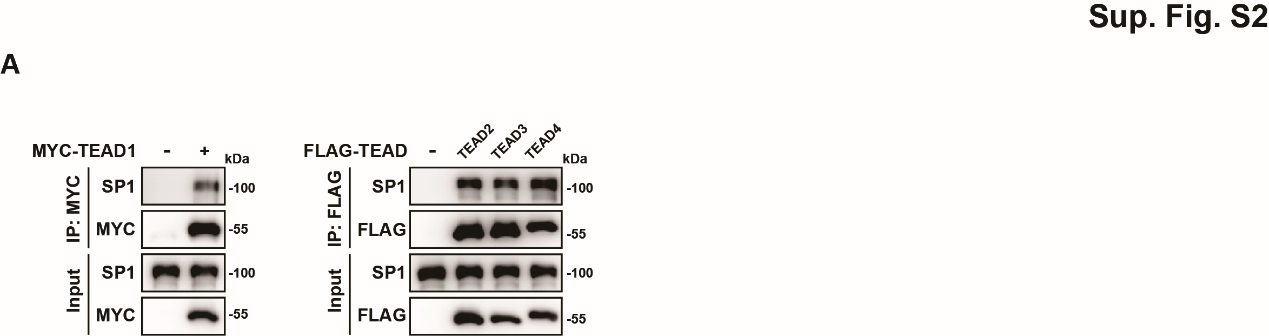


**Supplementary Figure S2. Related to Figure 2.**

1. Co-IP of exogenous Myc-TEAD1 and endogenous SP1 in HCT116 cells (left). Co-IP of exogenous FLAG-TEAD2/3/4 and endogenous SP1 in HCT116 cells (right).


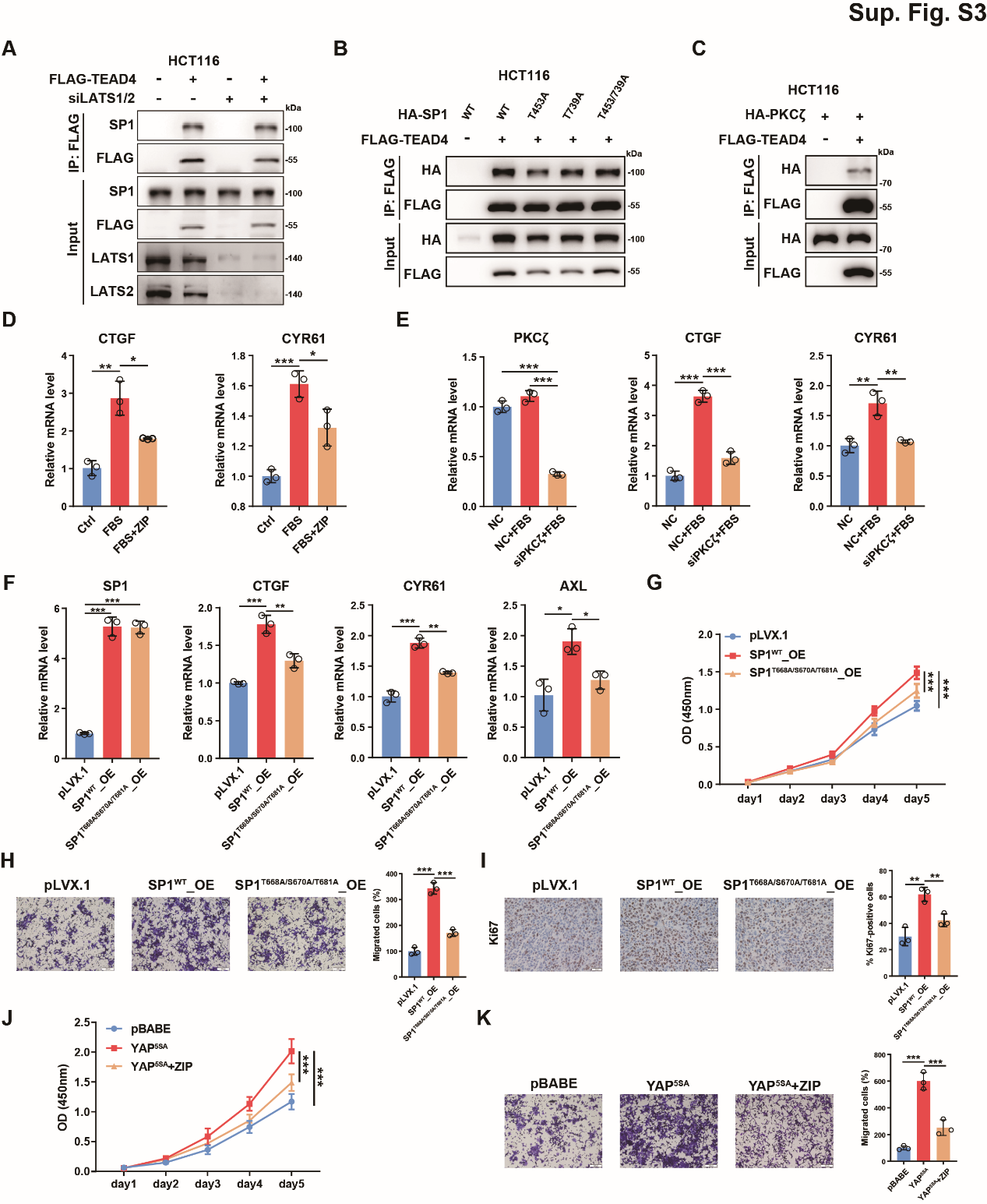


**Supplementary Figure S3. Related to Figure 3.**

1. Co-IP of exogenous FLAG-TEAD4 and endogenous SP1 in HCT116 cells with LATS1/2 knockdown.
2. Co-IP analysis of the interaction between TEAD4 and WT or mutant SP1 in HCT116 cells. T453 and T739 of SP1 are phosphorylated by ERKs.
3. Co-IP analysis of exogenous FLAG-TEAD4 and HA-PKCζ in HCT116 cells.
4. qPCR analysis of CTGF and CYR61 mRNA levels in HCT116 cells pretreated with 1 μM ZIP for 12 h and then stimulated with 10% serum for 15 min.
5. qPCR analysis of PKCζ, CTGF and CYR61 mRNA levels in the serum stimulation of PKCζ-knockdown HCT116 cells.
6. qPCR analysis of the SP1, CTGF, CYR61 and AXL mRNA levels in HCT116 cells overexpressing WT or the T668A/S670A/T681A SP1 fusion.
7. CCK8 assays of HCT116 cells overexpressing WT or T668A/S670A/T681A SP1.
8. Transwell assays of HCT116 cells overexpressing WT or T668A/S670A/T681A SP1.
9. Representative images and quantification of IHC staining for Ki67 in xenograft tumors derived from HCT116 cells overexpressing WT or T668A/S670A/T681A SP1.
10. CCK8 assays of HCT116 cells overexpressing constitutively YAP^5SA^ with or without ZIP (1 μM) treatment.
11. Transwell assays of HCT116 cells overexpressing constitutively YAP^5SA^ with or without ZIP (1 μM) treatment.


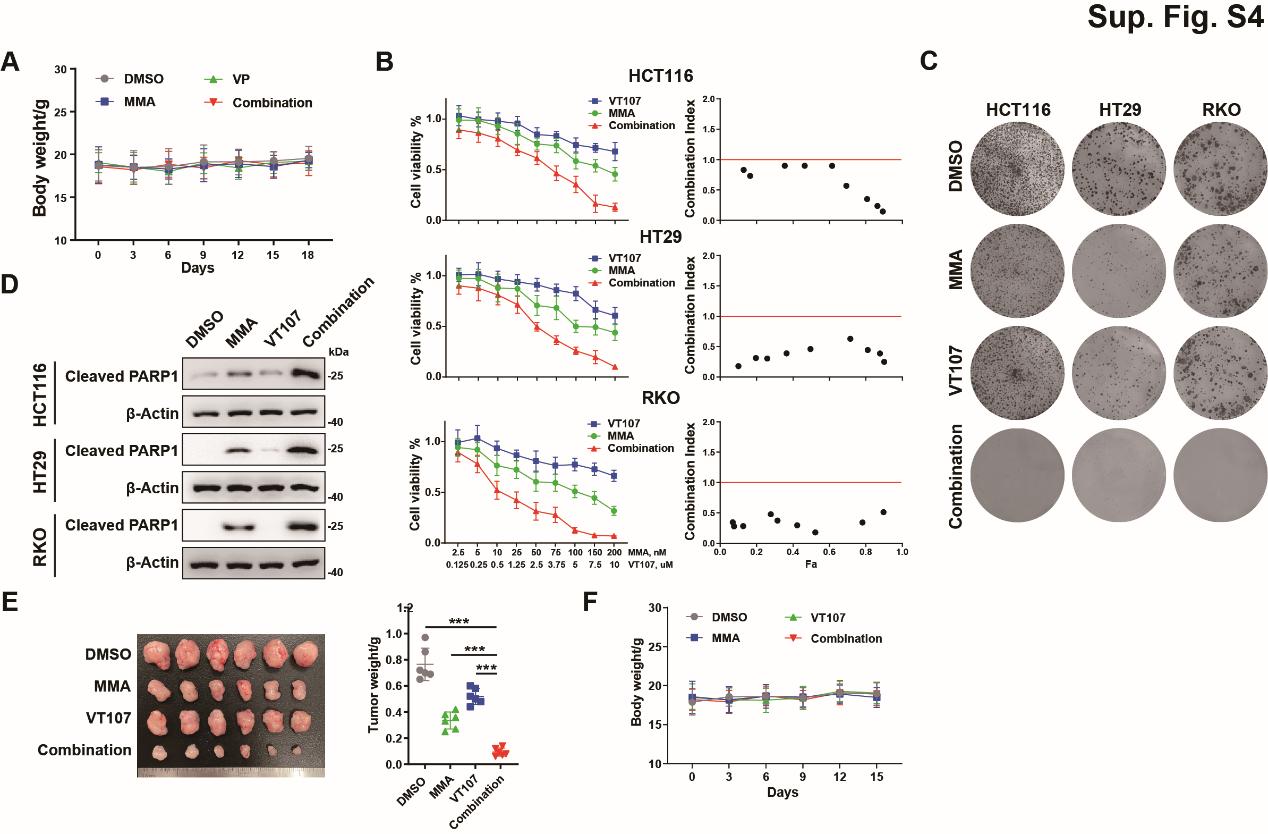


**Supplementary Figure S4. Related to Figure 4.**

1. The body weight curve of nude mice treated with vehicle, MMA, VP or the combination of MMA and VP (n = 6 mice per group).
2. Cell viability was assessed following 48 h of exposure to the indicated concentrations of MMA or VT107 alone or in combination in HCT116, HT29 and RKO cells (n=4 biologically independent samples per group). CI (combination index) values for the various combinations were calculated using CompuSyn. A CI<1.0 indicates a synergistic effect.
3. Colony formation assays showing the synergistic antitumor effects of the combination of MMA (100 nM) and VT107 (5 μM) on HCT116, HT29 and RKO cells.
4. Representative western blots of cleaved PARP1 in HCT116, HT29 and RKO cells treated with MMA (100 nM) or VT107 (5 μM) alone or in combination for 24 hours.
5. Representative images of HCT116 cell-derived xenografts harvested from nude mice treated with MMA and VT107 alone or in combination (n=6 mice per group) are shown. The xenograft tumors weights were measured for statistical analysis.
6. The body weight curve of nude mice treated with vehicle, MMA, VT107 or the combination of MMA and VT107 (n = 6 mice per group).


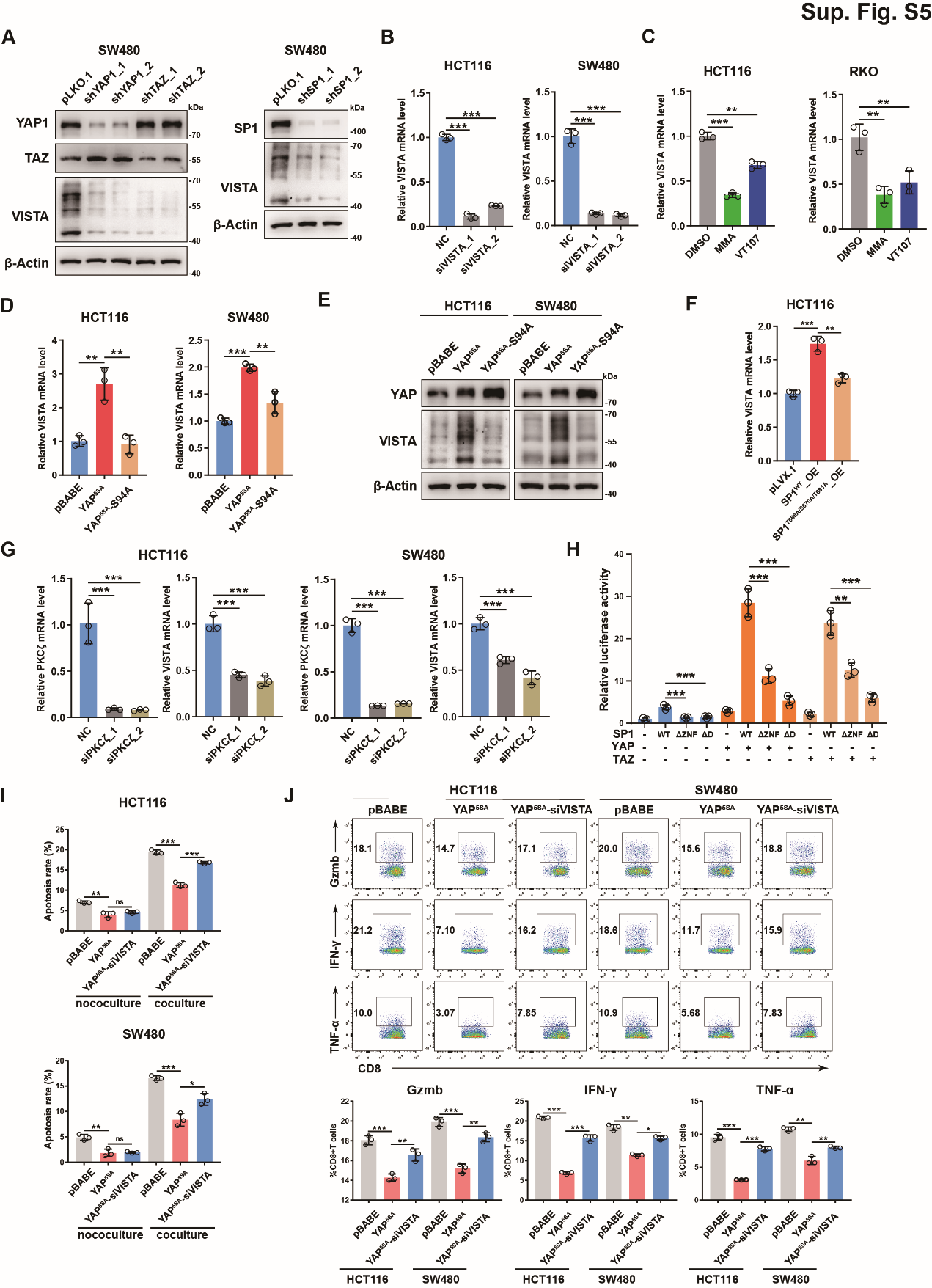


**Supplementary Figure S5. Related to Figure 5.**

1. Western blot analysis of the protein level of VISTA in SW480 cells with YAP, TAZ or SP1 knockdown.
2. qPCR analysis of the VISTA mRNA level in HCT116 and SW480 cells with VISTA knockdown.
3. qPCR analysis of the VISTA mRNA level in HCT116 and RKO cells treated with MMA (100 nM) or VT107 (5 μM) for 24 hours.
4. The mRNA expression of VISTA in HCT116 and SW480 cells overexpressing YAP^5SA^ or YAP^5SA^-S94A was detected via qPCR.
5. The protein expression of VISTA in HCT116 and SW480 cells overexpressing YAP^5SA^ or YAP^5SA^-S94A was detected via western blotting.
6. qPCR analysis of the VISTA mRNA level in HCT116 cells overexpressing WT or the T668A/S670A/T681A SP1.
7. qPCR analysis of the VISTA mRNA level in HCT116 and SW480 cells with PKCζ knockdown.
8. Relative luciferase activity of the VISTA promoter in HEK293T cells transfected with the indicated constructs of SP1, YAP and TAZ.
9. Cell apoptosis was analyzed by flow cytometry in YAP^5SA^-overexpressing HCT116 and SW480 cells with VISTA knockdown cultured alone or cocultured with CD8^+^ T cells.
10. CD8^+^ T cells were cocultured with YAP^5SA^-overexpressing HCT116 and SW480 cells with VISTA knockdown for 48 hours. Gzmb, IFN-γ and TNF-α expression was assessed via flow cytometry.


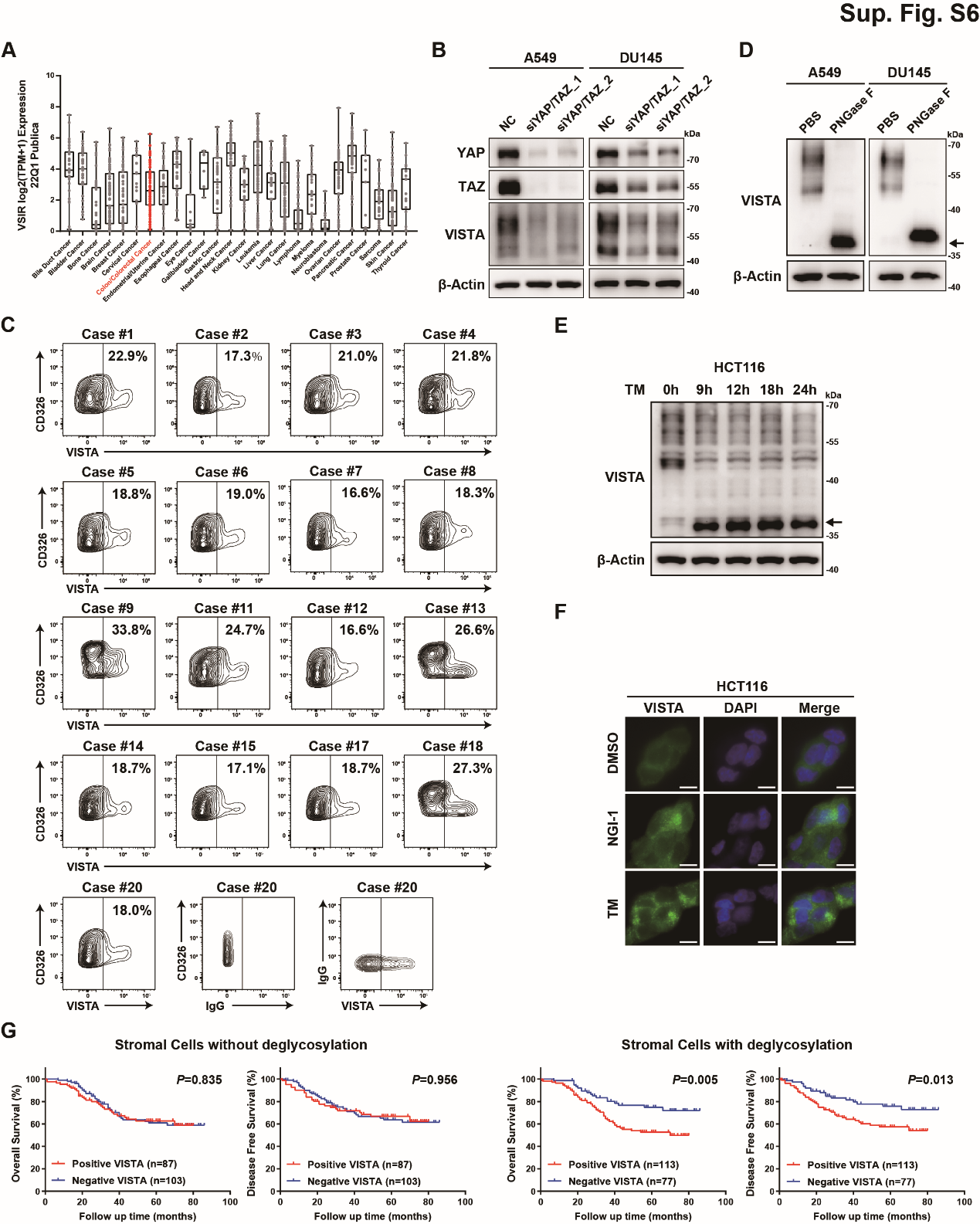


**Supplementary Figure S6. Related to Figure 6.**

1. VISTA mRNA levels in a panel of cancer cells. The data were extracted from the DepMap database.
2. Western blot analysis of the protein level of VISTA in A549 and DU145 cells with YAP and TAZ knockdown.
3. Flow cytometry analysis of VISTA protein expression in primary CRC tissues. Cells were gated for CD326 positivity to select epithelial cells, and the VISTA expression level was determined and analyzed in this subset compared to that in negative controls.
4. Lysates of A549 and DU145 cells were subjected to protein deglycosylation by PNGase F (5%) pretreatment, after which VISTA expression was detected via western blotting. The arrow indicates the nonglycosylated VISTA protein.
5. Western blot analysis of the protein level of VISTA in HCT116 cells pretreated with TM (1 μg/ml) for the indicated times. The arrow indicates nonglycosylated VISTA.
6. VISTA expression was detected by immunofluorescence in HCT116 cells pretreated with NGI-1 (10 μM) or TM (1 μg/ml) (scale bars=5 μm).
7. K‒M plots of OS and disease-free survival (DFS) of CRC patients stratified by the intensity of the VISTA signal in stromal cells. A CRC tissue array (TMA, n=190) was processed with or without deglycosylation by PNGase F (5%) pretreatment before regular IHC analysis.


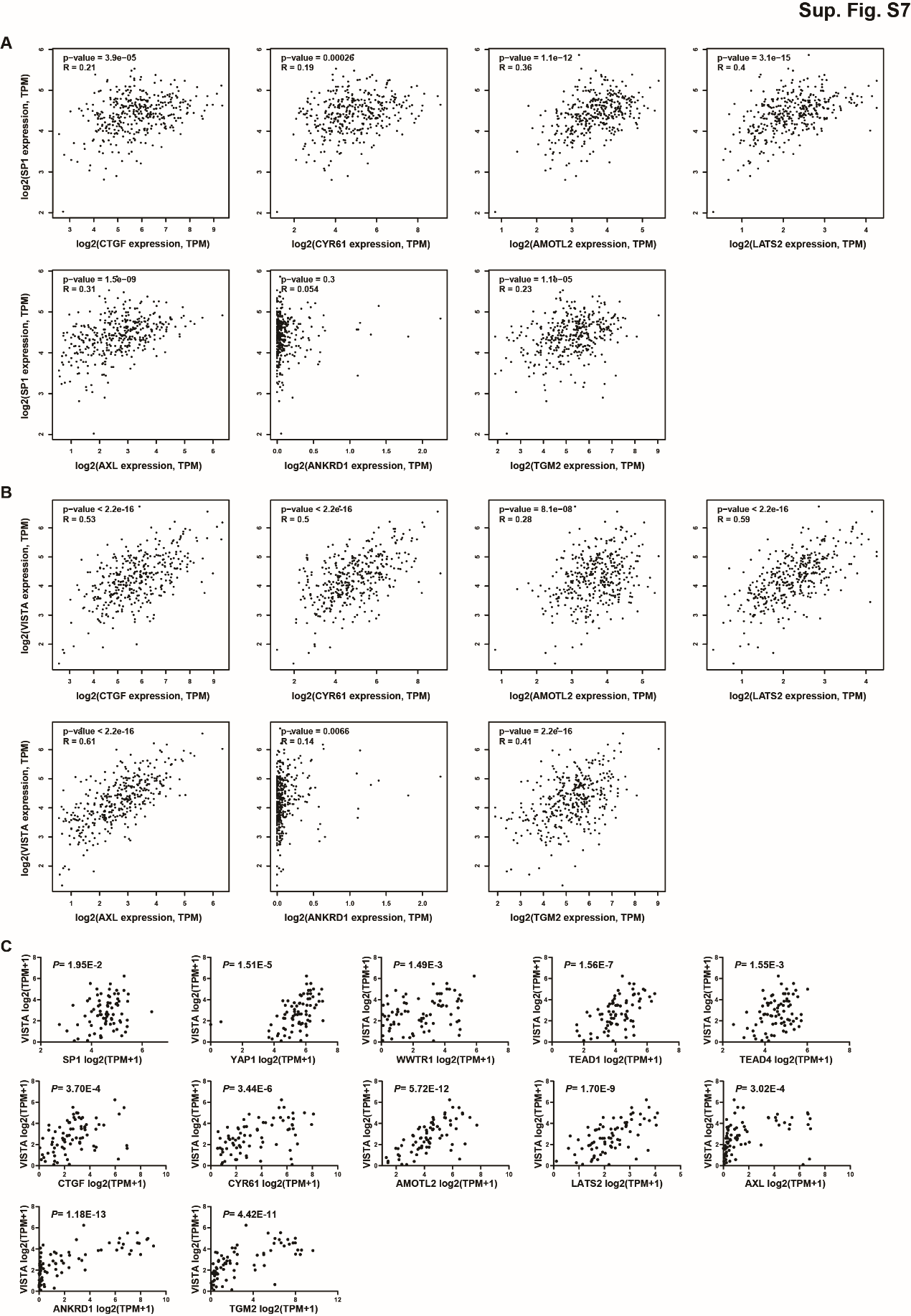


**Supplementary Figure S7. Related to Figure 7.**

1. Pearson correlation analysis showing a positive correlation between the SP1 mRNA level and the expression of YAP target genes (CTGF, CYR61, AMOTL2, LATS2, AXL, ANKRD1 and TGM2) in CRC. The data were extracted from the GEPIA2 database.
2. Pearson correlation analysis revealed a positive correlation between VISTA expression and the expression of YAP target genes (CTGF, CYR61, AMOTL2, LATS2, AXL, ANKRD1 and TGM2) in CRC. The data were extracted from the GEPIA2 database.
3. Pearson correlation analysis revealed positive correlations between VISTA expression and the mRNA levels of SP1, YAP, WWTR1, TEAD1, TEAD4 and YAP target genes (CTGF, CYR61, AMOTL2, LATS2, AXL, ANKRD1 and TGM2) in 76 CRC cell lines. The data were extracted from the DepMap database.

Supplementary Table 1. Sequence of shRNA and siRNA used in this study.

| shRNA/siRNA | Sequence (5’-3’) |
| --- | --- |
| siSP1_1 | GAAGGGAGGCCCAGGUGUA |
| siSP1_2 | GCUGGUGGUGAUGGAAUACAU |
| siPKCζ | POOL: CGCGTGATTGACCCTTTAACT/ CTGGTGCGGTTGAAGAAGAAT/ GCCTCCAGTAGACGACAAGAA |
| siVISTA_1 | CCCUGACUCUCCAAACUUUGA |
| siVISTA_2 | CACCAGCUACAGAUGCCAAAU |
| shSP1_1 | CCGGGAAGGGAGGCCCAGGTGTACTCGAGTACACCTGGGCCTCCCTTCTTTTTG |
| shSP1_2 | CCGGGCTGGTGGTGATGGAATACATCTCGAGATGTATTCCATCACCACCAGCTTTTTG |
| shYAP_1 | CCGGGACATCTTCTGGTCAGAGACTCGAGTCTCTGACCAGAAGATGTCTTTTTG |
| shYAP_2 | CCGGCTGGTCAGAGATACTTCTTCTCGAGAAGAAGTATCTCTGACCAGTTTTTG |
| shTAZ_1 | CCGGACGTTGACTTAGGAACTTTCTCGAGAAAGTTCCTAAGTCAACGTTTTTTG |
| shTAZ_2 | CCGGAGGTACTTCCTCAATCACACTCGAGTGTGATTGAGGAAGTACCTTTTTTG |

Supplementary Table 2. Sequence of real-time PCR primers used in this study.

| Gene | Forward Sequence 5’ to 3’ | Reverse Sequence 5’ to 3’ |
| --- | --- | --- |
| SP1 | GTGGAGGCAACATCATTGCTG | GCCACTGGTACATTGGTCACAT |
| YAP | ATCCCAGCACAGCAAATTCT | GGATTTTGAGTCCCACCAT |
| TAZ | GGCTGGGAGATGACCTTCAC | CTGAGTGGGGTGGTTCTGCT |
| CTGF | CCTGCAGGCTAGAGAAGCAG | TGGAGATTTTGGGAGTACGG |
| CYR61 | AAGAAACCCGGATTTGTGAG | GCTGCATTTCTTGCCCTTT |
| AXL | GTGGGCAACCCAGGGAATATC | GTACTGTCCCGTGTCGGAAAG |
| ANKRD1 | AGTAGAGGAACTGGTCACTGG | TGGGCTAGAAGTGTCTTCAGAT |
| LATS2 | ATGAGCTCCACTCTGCTCAATGTCACGG | GCAAGCTTCTCTACCAAGAATGAAAGAGCAT |
| AREG | CGAACCACAAATACCTGGCTA | TCCATTTTTGCCTCCCTTTT |
| CCND1 | GCTGCGAAGTGGAAACCATC | CCTCCTTCTGCACACATTTGAA |
| TGFB2 | CCAAAGGGTACAATGCCAAC | CAGATGCTTCTGGATTTATGGTATT |
| PKCζ | CTTACATTTCCTCATCCCGGAAG | TTCACCACTTTCATGGCGTAAA |
| VISTA | ACGCCGTATTCCCTGTATGTC | TTGTAGAAGGTCACATCGTGC |
| CTGF(ChIP) | GCCAATGAGCTGAATGGAGT | CAATCCGGTGTGAGTTGATG |
| CYR61(ChIP) | AGCAAACAGCTCACTGCCTT | ATGGTAGTTGGAGGGTCGTG |
| ANKRD1(ChIP) | ATGGCCTGCCACTTTGTTAC | TTTTCAGAACTGGGGTCTGG |
| Actin | GACCTGTACGCCAACACAG | CTCAGGAGGAGCAATGATC |

Supplementary Table 3. List of primary antibodies used in this study, and information on working dilutions of antibodies in Western blotting (WB), immunohistochemistry (IHC), immunofluorescence (IF) and flow cytometry (F).

| Antibody | Species | Source | Catalog# | Applications | Dilution |
| --- | --- | --- | --- | --- | --- |
| SP1 | Rabbit | ABclonal | A19649 | WB  IHC  IF | 1:1000  1:100  1:100 |
| YAP | Mouse | Santa Cruz Biotechnology | sc-101199 | WB  IHC  IF | 1:1000  1:100  1:100 |
| TAZ | Mouse | BD Bioscience | #560235 | IF | 1:100 |
| TAZ | Rabbit | Cell Signaling Technology | #8418 | WB | 1:1000 |
| TEAD4 | Mouse | Santa Cruz Biotechnology | sc-390578 | WB  IF | 1:1000  1:100 |
| Pan-TEAD | Rabbit | Cell Signaling Technology | #13295 | WB | 1:1000 |
| VISTA | Rabbit | Cell Signaling Technology | #64953 | WB  IHC | 1:1000  1:100 |
| FLAG | Rabbit | Cell Signaling Technology | #14793 | WB | 1:1000 |
| HA | Rabbit | Cell Signaling Technology | #3724 | WB | 1:1000 |
| Ki67 | Rabbit | Abcam | ab15580 | IHC | 1:100 |
| Cleaved PARP | Rabbit | Cell Signaling Technology | #5625 | WB  IHC | 1:1000  1:100 |
| β-Actin | Rabbit | Abcam | ab8226 | WB | 1:10000 |
| TNF-α | Mouse | Invitrogen | #17-7349-82 | F | 1:100 |
| IFNγ | Mouse | BD Bioscience | #562988 | F | 1:100 |
| Granzyme B | Mouse | Biolegend | #372208 | F | 1:100 |
| CD326 | Mouse | ThermoFisher | #53-9326-42 | F | 1:100 |
| VISTA | Mouse | ThermoFisher | #17-1088-42 | F | 1:100 |
